# Supplementary material for: Multiprofessional cross-site working between a level 1 and a level 3 neonatal unit: a retrospective cohort study
Source: BMJ Paediatr Open. 2022 Sep 22;6(1):e001581. doi: 10.1136/bmjpo-2022-001581 (PMC9511588; doi:10.1136/bmjpo-2022-001581)
Supplement: Author's manuscript [file bmjpo-2022-001581.draft_revisions.pdf]

BMJ Paediatrics Open

BMJ Paediatrics Open is committed to open peer review. As part of this commitment we make the peer review history of every article we publish publicly available.

When an article is published we post the peer reviewers' comments and the authors' responses online. We also post the versions of the paper that were used during peer review. These are the versions that the peer review comments apply to.

The versions of the paper that follow are the versions that were submitted during the peer review process. They are not the versions of record or the final published versions. They should not be cited or distributed as the published version of this manuscript.

BMJ Paediatrics Open is an open access journal and the full, final, typeset and author-corrected version of record of the manuscript is available on our site with no access controls, subscription charges or pay-per-view fees (http://bmjpaedsopen.bmj.com).

If you have any questions on BMJ Paediatrics Open's open peer review process please email info.bmjpo@bmj.com

BMJ Paediatrics Open

Multi-professional cross-site working between a level one and a level three neonatal unit: Impact on clinical outcomes

Journal:	*BMJ Paediatrics Open*
Manuscript ID	bmjpo-2022-001581
Article Type:	Original research
Date Submitted by the Author:	20-Jun-2022
Complete List of Authors:	Dassios, Theodore; King's College Hospital NHS Foundation Trust, Neonatal Intensive Care Unit; King's College London, Department of Women and Children's Health, School of Life Course Sciences, Faculty of Life Sciences and Medicine<br>Selvadurai, Lucksini; King's College Hospital NHS Foundation Trust, Neonatal Intensive Care Unit<br>Hickey, Ann; King's College Hospital NHS Foundation Trust, Neonatal Intensive Care Unit<br>Sleight, Elizabeth; King's College Hospital NHS Foundation Trust, Neonatal Intensive Care Unit<br>Long, Lisa; King's College Hospital NHS Foundation Trust, Department of Obstetrics and Gynaecology<br>Penna, Leonie; King's College Hospital NHS Foundation Trust, Department of Obstetrics and Gynaecology<br>Wallen-Mitchell, Vivette; King's College Hospital NHS Foundation Trust, Neonatal Intensive Care Unit<br>Bhat, Ravindra; King's College Hospital NHS Foundation Trust, Neonatal Intensive Care Unit<br>Greenough, Anne; King's College London, Women and Children's Health, School of Life Course Sciences; NIHR Biomedical Research Centre at Guy's and Saint Thomas' NHS Foundation Trust and King's College
Keywords:	Neonatology

SCHOLARONE™
Manuscripts

I, the Submitting Author has the right to grant and does grant on behalf of all authors of the Work (as defined in the below author licence), an exclusive licence and/or a non-exclusive licence for contributions from authors who are: i) UK Crown employees; ii) where BMJ has agreed a CC-BY licence shall apply, and/or iii) in accordance with the terms applicable for US Federal Government officers or employees acting as part of their official duties; on a worldwide, perpetual, irrevocable, royalty-free basis to BMJ Publishing Group Ltd ("BMJ") its licensees and where the relevant Journal is co-owned by BMJ to the co-owners of the Journal, to publish the Work in this journal and any other BMJ products and to exploit all rights, as set out in our licence.

The Submitting Author accepts and understands that any supply made under these terms is made by BMJ to the Submitting Author unless you are acting as an employee on behalf of your employer or a postgraduate student of an affiliated institution which is paying any applicable article publishing charge ("APC") for Open Access articles. Where the Submitting Author wishes to make the Work available on an Open Access basis (and intends to pay the relevant APC), the terms of reuse of such Open Access shall be governed by a Creative Commons licence – details of these licences and which Creative Commons licence will apply to this Work are set out in our licence referred to above.

Other than as permitted in any relevant BMJ Author's Self Archiving Policies, I confirm this Work has not been accepted for publication elsewhere, is not being considered for publication elsewhere and does not duplicate material already published. I confirm all authors consent to publication of this Work and authorise the granting of this licence.

Confidential: For Review Only

Multi-professional cross-site working between a level one and a level three neonatal unit: Impact on clinical outcomes

Theodore Dassios[1,2], Lucksini Selvadurai[1], Ann Hickey[1], Elizabeth Sleight[1], Lisa Long[3], Leonie Penna[3], Vivette Wallen-Mitchell[1], Ravindra Bhat[1], Anne Greenough[2,4].

[1]Neonatal Intensive Care Centre, King's College Hospital NHS Foundation Trust, London, United Kingdom;

[2]Department of Women and Children's Health, School of Life Course Sciences, Faculty of Life Sciences and Medicine, King's College London, London, United Kingdom;

[3]Department of Obstetrics and Gynaecology, King's College Hospital NHS Foundation Trust, London, United Kingdom;

[4]NIHR Biomedical Centre at Guy's and St Thomas NHS Foundation Trust and King's College London, London, United Kingdom

Address for correspondence: Dr Theodore Dassios, Neonatal Intensive Care Centre, 4th Floor Golden Jubilee Wing, King's College Hospital, Denmark Hill, London, SE5 9RS, United Kingdom. Tel: +44 0203 299 4563; E-mail: theodore.dassios@kcl.ac.uk

Word count of manuscript: 3,354

Key words: cross-site working, neonatal outcomes, special care baby unit

Confidential: For Review Only

ABSTRACT

Objective: To assess the impact on neonatal outcomes of cross-site working of multiple healthcare professional teams between a level three and a level one neonatal unit.

Design: Retrospective cohort study.

Setting: A level one neonatal unit in London.

Patients: All infants admitted to the neonatal unit, between 2010 and 2021.

Interventions: The clinical service was rearranged in 2014 with the introduction of cross-site working between the level one unit and a level three unit of neonatal doctors, nurses and allied healthcare professionals.

Main outcome measures: Admission of infants with a temperature less than 36°C, length of stay and time to first consultation by a senior team member.

Results: A total of 4,418 infants were admitted during the study period. The percentage of infants delivered at a gestation below 32 weeks was higher in the pre-cross-site period (8.9%) compared to the cross site period (3.6%, $p<0.001$). The percentage of infants with an Apgar score less than 8 at 10 minutes was higher in the pre-cross-site period (6.2%) compared to the cross-site period (3.4%, $p=0.001$). More infants were admitted with a temperature less than 36°C in the pre-cross site period (12.3%) compared to the cross site period (3.7%, $p<0.001$). The median (IQR) duration of time to first consultation by a senior team member was higher in the pre-cross-site period [1 (0.5 - 2.6) hours] compared to the cross-site period [0.5 (0.2 - 1.3) hours] ($p<0.001$). The median (IQR) length of stay was 4 (2-11) days in the pre-cross-site period and decreased to 2 (1-4) days in the cross-site period ($p<0.001$).

Conclusions: Cross-site working was associated with lower rates of admission hypothermia, shorter duration of stay and earlier first senior consultation.

Confidential: For Review Only

Key messages

What is already known on this topic:

Birth of high risk infants in a hospital with a tertiary neonatal unit is associated with better neonatal outcomes but neonatal care cannot be confined only to tertiary neonatal units.

What this study adds:

Cross-site working of neonatal doctors, nurses and allied healthcare professionals between a level three and a level one neonatal unit was associated with improved neonatal outcomes, such as lower rates of admission hypothermia, shorter duration of stay and earlier first senior consultation.

How this study might affect research, practice or policy:

Cross site working models could be adopted more widely by the neonatal community to improve the care of infants born in hospitals without tertiary neonatal facilities.

Confidential: For Review Only

INTRODUCTION

Neonatal care is stratified in three progressively more complex levels: level one, which refers to special care baby units, level two or local neonatal units and the highly specialised level three (tertiary) or neonatal intensive care units (NICU) [1]. Neonatal care in England is currently delivered in 161 neonatal units out of which only 44 are tertiary centres with the remaining 70% being non-tertiary [2]. The allocation of a neonatal unit as tertiary or non-tertiary is based on clearly specified criteria relating to the level of clinical support that can be offered, staff seniority and training [1, 3]. The tertiary units care for the sickest of infants and provide intensive care such as prolonged invasive ventilation, multiple modes of cardiorespiratory support and subspecialty services.

Neonatal complications arising in the first hours and days of life have significant consequences which may influence lifelong health and quality of life [4]. In some categories of infants, such as those born before 28 completed weeks of gestation and infants with antenatally-diagnosed major congenital anomalies, antenatal planning is mandated by national guidelines to ensure transfer of women to a tertiary centre for delivery (prenatal or in utero transfers) [2]. For these high-risk infants, birth in a hospital with a tertiary unit is associated with better neonatal outcomes such as lower odds of severe brain injury and higher odds of survival without severe brain injury compared to infants born in a hospital without a tertiary NICU and postnatally transferred to a tertiary NICU after stabilisation [5]. Birth in a tertiary unit is also associated with better outcomes in late or moderately born preterm infants [6].

Despite the aforementioned better outcomes associated with birth in a hospital with a tertiary neonatal unit, birth is a natural process and most babies are born healthy requiring little or no medical intervention. Neonatal care cannot be confined only to geographical areas with tertiary neonatal units and all pregnant women have a discussion around birth setting within

Confidential: For Review Only

their local area unless there are indications to deliver in a hospital with tertiary neonatal services. It is also important to note that infants at or close to term can be unexpectedly unwell and that premature birth cannot always be predicted resulting in unexpected births of sick and preterm infants in hospitals without tertiary neonatal services [7].

Better outcomes in tertiary NICUs result from the presence of medical and surgical subspecialties, increased funding, enhanced training and sophisticated equipment. Perhaps, however, the most important factor is the highly skilled staff who work in these units: the neonatal nurses, doctors and allied health professionals such as dieticians, physiotherapists and speech and language therapists. As not all infants can be delivered in hospitals with tertiary units, neonatal outcomes could, thus, be improved by allocating enhanced resources to non-tertiary neonatal units and cross-site working of skilled neonatal professionals that work primarily in a tertiary setting and can be partly deployed to non-tertiary units.

King's College Hospital NHS Foundation Trust (KCH) has a busy surgical and medical NICU and is located in South East London. KCH acquired in 2013 a level one neonatal unit at the Princess Royal University Hospital (PRUH) and gradually implemented cross-site working of doctors, nurses and allied health professionals. We hypothesised that the implementation of cross-site working between a level three and a level one neonatal unit would be associated with improved neonatal outcomes. Our aim was to test this hypothesis by comparing outcomes before and after the implementation of this model.

MATERIALS AND METHODS

Subjects and study design

A retrospective cohort study of all admissions to the Neonatal Unit at the PRUH between 1st January 2010 and 1st January 2022 was undertaken. The PRUH is located at Farnborough

Confidential: For Review Only

Common, Orpington, South East London, United Kingdom and is responsible for approximately 4,000 deliveries and 350-400 neonatal admissions per year [8]. The hospital has a level one neonatal unit with 10 cots. Level one units in the UK look after infants requiring special care and are suitable for deliveries at 32 weeks of gestation and above that are considered low risk and deliveries at 30-32 weeks of gestation subject to risk assessment. Infants requiring intensive care (for example invasive ventilation) or high dependency care (for example parenteral nutrition or short term invasive ventilation) are transferred to a higher level unit [1, 3]. Only infants who were born in the PRUH were included in this study; infants who were born in another hospital or whose main care was provided in another hospital and were transferred to the PRUH for continuation of care or pre-discharge, were not included as they were not thought to be representative of the care provided in the PRUH. If an infant had multiple admissions to the unit following referral from a local hospital or repatriation following specialist care, only the first episode was included in the analysis.

The level three unit of this study, was the tertiary neonatal intensive care centre of KCH NHS Trust, London, UK. KCH has a tertiary medical and surgical neonatal unit with 36 cots, with approximately 5,000 deliveries and 700 neonatal admissions per year. It serves a diverse community of over 1,000,000 in South East London and is part of the London Operational Network. Level 3 units in the United Kingdom are suitable for deliveries of infants at 27 weeks and below as well as infants that require high dependency and special care [2, 4].

The study was registered with the Clinical Governance Department of King's College Hospital NHS Foundation Trust. The Health Research Authority Toolkit of the National Health System, United Kingdom confirmed that the study was not considered as research and hence would not need regulatory approval by a research ethics committee.

Confidential: For Review Only

Data collected from the medical and nursing notes

Data were extracted from the BadgerNet Neonatal Electronic Patient Database (Clevermed, Edinburgh, UK). Mortality was defined as death before discharge from neonatal care [9]. The following data were collected: maternal age (years), administration of any antenatal steroids (yes/no), cord arterial pH, cord arterial pH <7.10 [10], gestational age (weeks), birth weight (kg), sex (male/female), Apgar score at 10 minutes, time of admission, admission temperature (^{o}C), admission blood glucose (mmol/L), time to first consultation by a senior member of staff (in hours), mechanical ventilation (yes/no), pneumothorax diagnosed on chest radiography (yes/no), whole body hypothermia (cooling) for hypoxic ischemic encephalopathy (yes/no), discharge home on supplemental oxygen (yes/no), postmenstrual age at discharge (weeks), total length of stay (days). The birthweight z-score was calculated using the UK-WHO preterm reference chart [11] and the Microsoft Excel add-in LMSgrowth (V.2.77; www.healthforchildren.co.uk). Hypothermia was defined as an admission temperature of less than 36.5 ^{o}C [12]. Hypoglycaemia was defined as a blood glucose concentration of less than 2.6 mmol/L [13].

Periods of implementation of cross-site working

Elements of the intervention

A comprehensive, gradual quality improvement programme was implemented that included up-skilling of the staff with cross-site education, training in newborn life support, simulation scenarios, regular multi-professional case review meetings and increased levels of staffing. Individual cases were regularly discussed for a second opinion with the senior neonatal Consultant on service at KCH which formed a separate rota of neonatal Consultants to the one at the PRUH. The neonatal team at KCH offered support and facilitated transfer for tertiary care when required. Although not covered in detail in this report, a similar

Confidential: For Review Only

programme was undertaken by the maternity team aiming to improve maternity outcomes across both sites.

Pre-cross-site period

The PRUH was acquired by KCH in October 2013. The first dedicated neonatal Consultant was in post in September 2014 and before this the paediatric Consultants covering the PRUH unit covered the neonatal unit as well as the paediatric wards and paediatric Accident and Emergency. The clinical cover for the neonatal unit in the pre-cross site period consisted of a Consultant covering both the paediatric and neonatal side and a middle grade doctor (Registrar) covering both the paediatric and neonatal sides of the service. For purposes of data entry consistency and to reflect current neonatal practice, the start point of the pre cross-site period was set in 2010. The pre-cross site period, thus, consisted of the chronological years 2010-2014 (five years).

Washout period

The cross-site working model was partly introduced in 2015 with complete implementation in late 2018 when a total of six cross-site neonatal Consultants were in post and formed a separate cross-site rota. In the years 2015-2018 a number of other changes were gradually introduced. Three fixed-term (one year) neonatal consultants covering only the PRUH neonatal unit were appointed in 2016 and 2017. A dedicated neonatal tier of middle grade doctors (registrars) who covered only the neonatal unit (and not the paediatric ward and the emergency department) was introduced in 2017. Four-week nursing placements of the PRUH nurses to KCH were introduced in 2017 and reciprocal nursing placements of KCH nurses to the PRUH were introduced in 2018. Cross-site clinical guidelines were also gradually introduced and implemented in 2017 and 2018. The washout period between 2015 and 2018 was deemed essential as the aforementioned changes were introduced during this

https://mc.manuscriptcentral.com/bmjpo

Confidential: For Review Only

period and not all Consultant posts were consistently filled. The washout period thus corresponded to the chronological years of 2015-2018 (four years).

Cross-site period

A complete rota of cross-site neonatal Consultants that were working across both KCH and the PRUH was fully implemented by 2019. A cross-site Neonatal Lead Nurse for both sites was appointed in 2019. Nursing placements of the PRUH nurses to KCH were achieved with 80% of senior nurses undertaking a placement/rotation by 2019. The cross-site period thus, consisted of the chronological years 2019-2021 (three years). The year 2022 was not included as the data collection for this projected started in April 2022. The total number of beds did not change during the study period.

Statistical analysis

The data were tested for normality with the Kolmogorov-Smirnov test, were found to be not normally distributed and were thus presented as median (interquartile range). The Mann Whitney U test was used for comparisons of variables before and after the implementation of the cross-site working model. Binary variables were compared between the two periods with the chi square test. The temporal evolution of the length of stay in days, the time to first consultation by a senior member of the team in hours and the admission temperature in ^{o}C were presented in boxplots with each year presented as a separate boxplot.

Statistical analysis was performed using SPSS V.26.0 software.

Confidential: For Review Only

RESULTS

A total of 4,418 infants were admitted to the neonatal unit at the PRUH during the study period. A total of 303 infants were repatriated to the PRUH having been born in another hospital and were thus excluded from the analysis. In the pre-cross-site period, 1,299 infants and in the cross-site period 1,052 infants were born and admitted in the neonatal unit of the PRUH. In the washout period a total of 1,765 infants were born and admitted in the neonatal unit (data not presented).

The characteristics and outcomes of the included infants in the two study periods are presented in table 1. The percentage of infants delivering at the PRUH at a gestation below 32 weeks was higher in the pre-cross-site period compared to the cross site period ($p<0.001$). The percentage of infants with an Apgar score of less than 8 at 10 minutes of life, was higher in the pre-cross-site period compared to the cross site period ($p=0.001$). More infants were admitted with hypothermia and an admission temperature of less than 36°C in the pre-cross site period compared to the cross site period ($p<0.001$). Furthermore, the median duration of time to the first consultation by a member of the admitting team was higher in the pre-cross-site period compared to the cross-site period ($p<0.001$) with more parents receiving the first consultation/update after four hours from the time of admission compared to the cross-site period ($p<0.001$). The median length of stay was higher in the pre-cross-site period compared to the cross-site period ($p<0.001$). The incidence of an arterial pH of less than 7.1 was higher in the infants in whom cord gases were performed in the pre cross-site period compared to the cross-site period ($p<0.001$). There were no significant differences in the administration of antenatal steroids, admission hypoglycaemia and therapeutic cooling between the two study periods (table 1).

Confidential: For Review Only

Table 1: Characteristics and outcomes of the admitted infants in the two study periods. Median (IQR) or *N* (%)

	Pre cross-site *N*=1,299	Cross-site *N*=1,052	p value
Maternal age (years)	32 (27 – 35)	32 (28 – 36)	0.016
Antenatal steroids	451 (34.7)	310 (29.5)	0.127
Cord arterial pH	7.25 (7.12 – 7.32)	7.27 (7.19 – 7.32)	0.003
Cord arterial pH<7.1	97 of 516 (18.8)	57 of 592 (9.6)	<0.001
Gestational age (weeks)	37.1 (34.1 – 39.9)	38.8 (35.9– 40.3)	<0.001
Gestation < 32 weeks	116 (8.9)	38 (3.6)	<0.001
Birth weight (kg)	2.76 (2.01 – 3.45)	3.14 (2.50 – 3.61)	<0.001
Birth weight z score	-0.15 (-0.83 – 0.53)	-0.06 (-0.77 – 0.60)	0.019
Male sex	773 (59.5)	589 (56.0)	0.217
Apgar at 10 minutes <8	81 (6.2)	36 (3.4)	0.001
Admission hypothermia	346 (26.6)	180 (17.1)	<0.001
Admission temperature<36°C	160 (12.3)	39 (3.7)	<0.001
Admission hypoglycaemia	327 (25.1)	245 (23.3)	0.193
Time to first consultation (h)	1.0 (0.5 – 2.6)	0.5 (0.2 – 1.3)	<0.001
Time to first consultation >4h	134 (10.3)	57 (5.4)	<0.001
Pneumothorax	18 (1.4)	19 (1.8)	0.528
Cooling	17 (1.3)	14 (1.3)	0.961
Home oxygen	129 (9.9)	146 (13.8)	<0.001
Mortality	12 (0.9)	1 (0.1)	<0.001
Length of stay (days)	4 (2 – 11)	2 (1 – 4)	<.0.001
Postmenstrual age at discharge (wks)	38.1 (36.0 – 40.2)	39.1 (36.5 – 40.4)	<0.001
Weight at discharge	2.66 (2.04 – 3.39)	3.08 (2.42 – 3.58)	<0.001

The evolution of the length of stay, time to first consultation and admission temperature over the years of the study period is presented in figure 1.

https://mc.manuscriptcentral.com/bmjpo

Confidential: For Review Only

DISCUSSION

We have demonstrated that cross-site working between a level three and a level one neonatal unit was associated with improved neonatal outcomes including less admission hypothermia, shorter duration of stay and more timely first consultation by senior members of the team.

Our results highlight a successful method in which a level-one unit can maintain and improve quality standards by working together with a tertiary level. This has implications within networks of neonatal care, as such models could be adopted more widely to improve the care of infants born in hospitals without tertiary neonatal facilities. Of note, the results of our study relate to a unit with a workload which is higher compared to other level one units in London in the same study periods. A recent survey of neonatal units reported live births ranging from two to three thousands per year in other level one units in London [14]. Our method thus could be feasible in the lower-volume level one units.

We could not compare our study results with studies reporting on interventions to improve neonatal outcomes on level one units, as to the best of our knowledge there are no other such studies in the literature. Cross-site working between a level three and a level one unit is not the only potential model of care that could theoretically improve neonatal outcomes in level one units. Other described models include the development of the roles of the enhanced or advanced neonatal nurse practitioners, physician associates or associate specialists [14]. These, however, are mostly recent developments with a paucity of published evidence on the impact of these models on neonatal outcomes. A number of other factors can affect neonatal outcomes such as the impact of culture and morale on the workforce, investment in clinical supervision and training, implementation of quality improvement projects and integrated family development care. We could not capture these outcomes, as they are not quantified and documented in the neonatal data entry software [14].

Confidential: For Review Only

Certain of the improved outcomes that we have reported following the introduction of the cross-site working model, might also be explained by improved maternity care which might be the product of cross-site working in the maternity services. Such improved outcomes include a smaller percentage of infants born with a low cord pH and a lower number of infants born at less than 32 weeks of gestation, signalling more efficient in utero transfers of women at risk. The consistent presence of neonatal Consultants who did not have commitments to other paediatric services facilitated better and more frequent communication and interaction between the neonatal team and the obstetric and midwifery teams. Furthermore, improved admission temperature might also be related to better maternity care in the delivery suite, obstetric theatres and postnatal wards. The non-significant increase in the administration of antenatal steroids might be explained by the smaller percentage of infants that delivered prematurely in the PRUH and would thus qualify for this intervention [15]. The smaller percentage of infants that had a lower Apgar score at 10 minutes might be related to improved resuscitation at birth, but could also be partly explained by the smaller number of infants that were born with a lower cord arterial pH. We recognise, thus, that maternity improvement clearly played a major part in our improved neonatal outcomes. The majority, though, of the reported outcomes in our study are direct quality indicators of neonatal care and could be partly attributed to improved multi-professional neonatal care [1].

Our study has significant financial implications. Neonatal services are costly as they require expensive equipment, consumables and employment of a skilled multi-professional workforce which operates 24 hours a day. Neonatal units have been reported to have an incomplete understanding of the costs of running the service [16]. We did not perform a separate financial analysis, as all care days were at the same level and cost. We reported, however, that the median length of stay was halved, which corresponds to a cost reduction of a similar magnitude. It follows that if this intervention was implemented on a wider scale, it could be associated with significant cost reductions. Daily charges for neonatal care vary across different Trusts, with little consensus on the basis on which these charges are

Confidential: For Review Only

determined, but an average cost of special care per day is approximately 500 GBP [17]. At a single level one unit, thus, with 400 admissions per year the cost saving would be approximately 100,000 GBP per year. We do not present in this paper a full financial analysis of the intervention but these numbers should be taken in the context of the relevant staffing and equipment expenditure. Irrespective of the financial aspects, it is important to remember that decreased length of stay and earlier discharge by senior clinicians coupled with appropriate outpatient follow-up arrangements promote normal family life in a home environment with enhanced quality of sleep, less exposure to infection risks [18] and improved neurodevelopment [19].

Interestingly, the introduction of a cross-site model was not associated with a reduction of the total number of admissions per year. This might be explained by the fact that newborn infants constitute an already high-risk patient population. According to the World Health Organisation in 2019, 47% of all deaths in under five year olds occurred in the newborn period [20]. Previous neonatal studies have focused on improving outcomes of premature infants and more so the extremely premature ones. It is interesting, however, to note that the vast majority of births are term or late preterm and neonatal admissions are commonly infants who require a brief episode of care. Our study is thus relevant to a wider population than the results of the specialised studies of extreme prematurity and more applicable to the wider public.

Our study has strengths and some limitations. This is, to our knowledge, the first neonatal study to assess the effect of cross-site working on neonatal outcomes. We used a large population of infants that were cared for on the same unit minimising potential differences that relate to different clinical practices or standards of care between units. The applicability of our study lies in that the improved neonatal outcomes following the introduction of cross-site working, if confirmed by larger and more diverse-population studies, could strengthen the argument of implementing a cross-site neonatal model of care. We acknowledge as a limitation that it is impossible to completely separate the relative contribution of cross-site

Confidential: For Review Only

working to improved outcomes from the overall global tendency for better outcomes in neonatal care. For example, there has been an international drive to recognise and prevent neonatal hypothermia following the recognition of the detrimental effects of this complication on neonatal outcomes [21]. The International Liaison Committee on Resuscitation recommended in a consensus statement in 2015 that the admission temperature of newborn infants should be maintained between 36.5 and 37.5 ^{o}C after birth, during stabilisation and admission, as temperature was a strong predictor of mortality and morbidity [22]. Other outcomes though, such as earlier consultation and discharge and timely in utero transfer, can be predominantly attributed to the implementation of novel workforce models such as cross-site working. Our study cannot establish a clear causal link between the introduction of cross-site working and improved neonatal outcomes as it is retrospective and spans over twelve years. Nevertheless, it is important to share with the neonatal community our experience of this intervention which could have significant policy implications.

In conclusion, cross-site working between a level three and a level one neonatal unit was associated with improved neonatal outcomes relevant to level one neonatal care, such as a reduction in admission hypothermia, shorter length of stay and more timely first consultation by senior members of the team.

Confidential: For Review Only

FIGURE LEGENDS

Figure 1: Evolution of the length of stay (a), time to first consultation (b) and admission temperature (c) over the study period. The horizontal lines in the boxes represent the lower quartile, median and upper quartile values of the presented parameters and the whiskers the minimum and maximum values. The whiskers do not include the outliers, defined as values more than one and a half box lengths from the median which are presented as open circles. The whiskers do not include the extreme outliers, defined as values more than three box lengths from the median which are presented as asterisks.

Confidential: For Review Only

Funding statement: This project was partially supported by King's College London 2018 Medical Research Council Confidence in Concept Award through the King's Health Partners' Research and Development Challenge Fund (Grant code: MC_PC_18052).

Competing interests statement: The authors have no competing interests to declare.

Author's contribution: TD conceived the study, contributed to data analysis and interpretation and wrote the first version of the manuscript. LS collected the data and contributed to data analysis and writing the manuscript. AH led the implementation of the intervention, contributed to study design and critically revised the manuscript. ES lead the implementation of the intervention and critically revised the manuscript. LL contributed to the study design and critically revised the manuscript. LP contributed to the study design and critically revised the manuscript. VWM contributed to the study design and critically revised the manuscript. RB led the implementation of the intervention, contributed to study design and critically revised the manuscript. AG contributed to project supervision, funding acquisition and extensively revised and edited the manuscript. All authors have read and agreed to the published version of the manuscript.

Data sharing statement: Data is available to share upon request.

Confidential: For Review Only

References

1. NHS. Toolkit for High Quality Neonatal Services. London, UK2009.

2. Improvement NEaN. Implementing the Recommendations of the Neonatal Critical Care Transformation Review. In: Improvement NEaN, editor. 2019.

3. BAPM. Service standards for hospitals providing neonatal care. 3rd Edition. 2010.

4. Luu TM, Rehman Mian MO, Nuyt AM. Long-term impact of preterm birth: Neurodevelopmental and physical health outcomes. *Clin Perinatol* 2017;44:305-14.

5. Helenius K, Longford N, Lehtonen L, et al. Association of early postnatal transfer and birth outside a tertiary hospital with mortality and severe brain injury in extremely preterm infants: observational cohort study with propensity score matching. *BMJ* 2019;367:l5678.

6. Boyle EM, Johnson S, Manktelow B, et al. Neonatal outcomes and delivery of care for infants born late preterm or moderately preterm: a prospective population-based study. *Arch Dis Child Fetal Neonatal Ed* 2015;100:F479-85.

7. Suff N, Story L, Shennan A. The prediction of preterm delivery: What is new? *Sem Fetal Neonatal Med* 2019;24:27-32.

8.https://www.ons.gov.uk/aboutus/transparencyandgovernance/freedomofinformationfoi/hospitalstatistics: Office for National Statistics; [cited 2022 03/05].

9. Smith LK, Draper ES, Manktelow BN, et al. Socioeconomic inequalities in survival and provision of neonatal care: population based study of very preterm infants. *BMJ* 2009;339:b4702.

10. Yeh P, Emary K, Impey L. The relationship between umbilical cord arterial pH and serious adverse neonatal outcome: analysis of 51,519 consecutive validated samples. *BJOG* 2012;119:824-31.

11. Wright CM, Booth IW, Buckler JM, et al. Growth reference charts for use in the United Kingdom. *Arch Dis Child* 2002;86:11-4.

Confidential: For Review Only

12. Laptook AR, Bell EF, Shankaran S, et al. Admission temperature and associated mortality and morbidity among moderately and extremely preterm infants. J Pediatr 2018;192:53-9 e2.

13. BAPM. Identification and management of neonatal hypoglycaemia in the full term Infant. 2017.

14. Mitra T, Bramwells L. Exploring new ways of working in the neonatal unit. London: London Neonatal Operational Delivery Network; 2017.

15. Chawla S, Natarajan G, Shankaran S, et al. Association of neurodevelopmental outcomes and neonatal morbidities of extremely premature infants with differential exposure to antenatal steroids. *JAMA Pediatr* 2016170:1164-72.

16. Accounts HoCCoP. Caring for Vulnerable Babies: The reorganisation of neonatal services in England. London House of Commons; 2008.

17. Dassios T, Greenough A, Leontiadi S, et al. Admissions for hypoglycaemia after 35 weeks of gestation: perinatal predictors of cost of stay. *J Matern Fetal Neonatal Med* 2019;32:448-54.

18. Jones E, Stewart F, Taylor B, et al. Early postnatal discharge from hospital for healthy mothers and term infants. *Cochrane Database Syst Rev* 2021;6:CD002958.

19. Losbar JL, Montjaux N, Ehlinger V, et al. Early discharge and hospital-assisted home care is associated with better neurodevelopmental outcome in preterm infants. *Early Hum Dev* 2021;161:105451.

20. WHO. Newborns: improving survival and well-being: WHO 2020 [cited 2022 02/05]. Available from: https://www.who.int/news-room/fact-sheets/detail/newborns-reducing-mortality#:~:text=Causes,within%20the%20first%2024%20hours.

21. Lunze K, Bloom DE, Jamison DT, et al. The global burden of neonatal hypothermia: systematic review of a major challenge for newborn survival. *BMC Med* 2013;11:24.

22. Perlman JM, Wyllie J, Kattwinkel J, et al. Part 7: Neonatal Resuscitation: 2015 International Consensus on Cardiopulmonary Resuscitation and Emergency Cardiovascular Care Science With Treatment Recommendations. *Circulation* 2015;132:S204-41.

Confidential: For Review Only

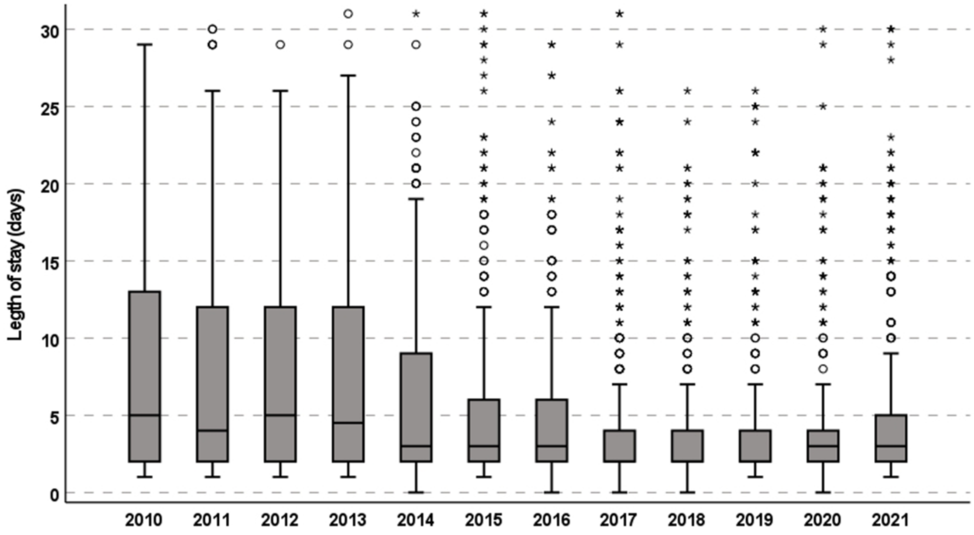

Figure 1: Evolution of the length of stay (a), time to first consultation (b) and admission temperature (c) over the study period. The horizontal lines in the boxes represent the lower quartile, median and upper quartile values of the presented parameters and the whiskers the minimum and maximum values. The whiskers do not include the outliers, defined as values more than one and a half box lengths from the median which are presented as open circles. The whiskers do not include the extreme outliers, defined as values more than three box lengths from the median which are presented as asterisks.

300x177mm (300 x 300 DPI)

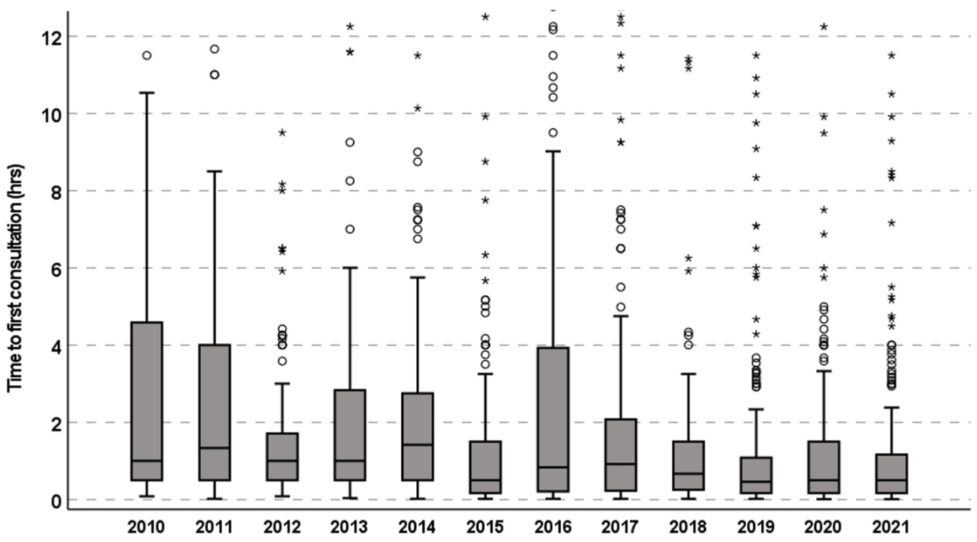

Figure 1: Evolution of the length of stay (a), time to first consultation (b) and admission temperature (c) over the study period. The horizontal lines in the boxes represent the lower quartile, median and upper quartile values of the presented parameters and the whiskers the minimum and maximum values. The whiskers do not include the outliers, defined as values more than one and a half box lengths from the median which are presented as open circles. The whiskers do not include the extreme outliers, defined as values more than three box lengths from the median which are presented as asterisks.

300x177mm (300 x 300 DPI)

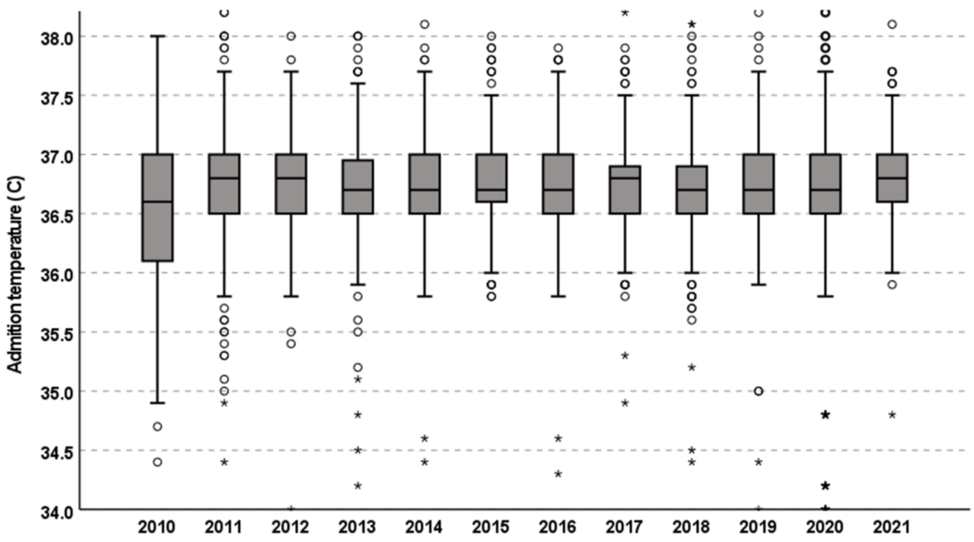

Figure 1: Evolution of the length of stay (a), time to first consultation (b) and admission temperature (c) over the study period. The horizontal lines in the boxes represent the lower quartile, median and upper quartile values of the presented parameters and the whiskers the minimum and maximum values. The whiskers do not include the outliers, defined as values more than one and a half box lengths from the median which are presented as open circles. The whiskers do not include the extreme outliers, defined as values more than three box lengths from the median which are presented as asterisks.

300x177mm (300 x 300 DPI)

BMJ Paediatrics Open

Multi-professional cross-site working between a level one and a level three neonatal unit: a retrospective cohort study

Journal:	*BMJ Paediatrics Open*
Manuscript ID	bmjpo-2022-001581.R1
Article Type:	Original research
Date Submitted by the Author:	22-Jul-2022

Complete List of Authors:	Dassios, Theodore; King's College Hospital NHS Foundation Trust, Neonatal Intensive Care Unit; King's College London, Department of Women and Children's Health, School of Life Course Sciences, Faculty of Life Sciences and Medicine<br>Selvadurai, Lucksini; King's College Hospital NHS Foundation Trust, Neonatal Intensive Care Unit<br>Hickey, Ann; King's College Hospital NHS Foundation Trust, Neonatal Intensive Care Unit<br>Sleight, Elizabeth; King's College Hospital NHS Foundation Trust, Neonatal Intensive Care Unit<br>Long, Lisa; King's College Hospital NHS Foundation Trust, Department of Obstetrics and Gynaecology<br>Penna, Leonie; King's College Hospital NHS Foundation Trust, Department of Obstetrics and Gynaecology<br>Wallen-Mitchell, Vivette; King's College Hospital NHS Foundation Trust, Neonatal Intensive Care Unit<br>Bhat, Ravindra; King's College Hospital NHS Foundation Trust, Neonatal Intensive Care Unit<br>Greenough, Anne; King's College London, Women and Children's Health, School of Life Course Sciences; NIHR Biomedical Research Centre at Guy's and Saint Thomas' NHS Foundation Trust and King's College

Keywords:	Neonatology

SCHOLARONE™
Manuscripts

I, the Submitting Author has the right to grant and does grant on behalf of all authors of the Work (as defined in the below author licence), an exclusive licence and/or a non-exclusive licence for contributions from authors who are: i) UK Crown employees; ii) where BMJ has agreed a CC-BY licence shall apply, and/or iii) in accordance with the terms applicable for US Federal Government officers or employees acting as part of their official duties; on a worldwide, perpetual, irrevocable, royalty-free basis to BMJ Publishing Group Ltd ("BMJ") its licensees and where the relevant Journal is co-owned by BMJ to the co-owners of the Journal, to publish the Work in this journal and any other BMJ products and to exploit all rights, as set out in our licence.

The Submitting Author accepts and understands that any supply made under these terms is made by BMJ to the Submitting Author unless you are acting as an employee on behalf of your employer or a postgraduate student of an affiliated institution which is paying any applicable article publishing charge ("APC") for Open Access articles. Where the Submitting Author wishes to make the Work available on an Open Access basis (and intends to pay the relevant APC), the terms of reuse of such Open Access shall be governed by a Creative Commons licence – details of these licences and which Creative Commons licence will apply to this Work are set out in our licence referred to above.

Other than as permitted in any relevant BMJ Author's Self Archiving Policies, I confirm this Work has not been accepted for publication elsewhere, is not being considered for publication elsewhere and does not duplicate material already published. I confirm all authors consent to publication of this Work and authorise the granting of this licence.

Confidential: For Review Only

Multi-professional cross-site working between a level one and a level three neonatal unit: a retrospective cohort study

Theodore Dassios[1,2], Lucksini Selvadurai[1], Ann Hickey[1], Elizabeth Sleight[1], Lisa Long[3], Leonie Penna[3], Vivette Wallen-Mitchell[1], Ravindra Bhat[1], Anne Greenough[2,4].

[1]Neonatal Intensive Care Centre, King's College Hospital NHS Foundation Trust, London, United Kingdom;

[2]Department of Women and Children's Health, School of Life Course Sciences, Faculty of Life Sciences and Medicine, King's College London, London, United Kingdom;

[3]Department of Obstetrics and Gynaecology, King's College Hospital NHS Foundation Trust, London, United Kingdom;

[4]NIHR Biomedical Centre at Guy's and St Thomas NHS Foundation Trust and King's College London, London, United Kingdom

Address for correspondence: Dr Theodore Dassios, Neonatal Intensive Care Centre, 4th Floor Golden Jubilee Wing, King's College Hospital, Denmark Hill, London, SE5 9RS, United Kingdom. Tel: +44 0203 299 4563; E-mail: theodore.dassios@kcl.ac.uk

Confidential: For Review Only

Word count of manuscript: 3,808

Key words: cross-site working, neonatal outcomes, special care baby unit

ABSTRACT

Objective: To assess the association of short-term neonatal outcomes with cross-site working of multiple healthcare professional teams between a level three and a level one neonatal unit.

Design: Retrospective cohort study.

Setting: A level one neonatal unit in London.

Patients: All infants admitted to the neonatal unit, between 2010 and 2021.

Interventions: The clinical service was rearranged in 2014 with the introduction of cross-site working between the level one unit and a level three unit of neonatal doctors, nurses and allied healthcare professionals.

Main outcome measures: Admission of infants with a temperature less than 36°C, length of stay and time to first consultation by a senior team member.

Results: A total of 4,418 infants were admitted during the study period. The percentage of infants delivered at a gestation below 32 weeks was higher in the pre cross-site period (8.9%) compared to the cross site period (3.6%, p<0.001). The percentage of infants with an Apgar score less than 8 at 10 minutes was higher in the pre cross-site period (6.2%) compared to the cross-site period (3.4%, p=0.001). More infants were admitted with a temperature less than 36°C in the pre-cross site period (12.3%) compared to the cross site period (3.7%, p<0.001). The median (IQR) duration of time to first consultation by a senior team member was higher in the pre cross-site period [1 (0.5 - 2.6) hours] compared to the cross-site period [0.5 (0.2 - 1.3) hours] (p<0.001). The median (IQR) length of stay was 4 (2-11) days in the pre cross-site period and decreased to 2 (1-4) days in the cross-site period (p<0.001).

Conclusions: Cross-site working was associated with lower rates of admission hypothermia, shorter duration of stay and earlier first senior consultation.

Confidential: For Review Only

Key messages

What is already known on this topic:

Birth of high risk infants in a hospital with a tertiary neonatal unit is associated with better short-term neonatal outcomes but neonatal care cannot be confined only to tertiary neonatal units.

What this study adds:

Cross-site working of neonatal doctors, nurses and allied healthcare professionals between a level three and a level one neonatal unit was associated with improved short-term neonatal outcomes, such as lower rates of admission hypothermia, shorter duration of stay and earlier first senior consultation.

How this study might affect research, practice or policy:

Cross site working models could be adopted more widely by the neonatal community to improve the care of infants born in hospitals without tertiary neonatal facilities.

Confidential: For Review Only

INTRODUCTION

In the United Kingdom, neonatal care is stratified in three progressively more complex levels: level one, which refers to special care baby units, level two or local neonatal units and the highly specialised level three (tertiary) or neonatal intensive care units (NICU) [1]. Neonatal care in England is currently delivered in 161 neonatal units out of which only 44 are tertiary centres with the remaining 70% being non-tertiary [2]. The allocation of a neonatal unit as tertiary or non-tertiary is based on clearly specified criteria relating to the level of clinical support that can be offered, staff seniority and training [1, 3]. The tertiary units care for the sickest of infants and provide intensive care such as prolonged invasive ventilation, multiple modes of cardiorespiratory support and subspecialty services.

Neonatal complications arising in the first hours and days of life have significant consequences which may influence lifelong health and quality of life [4]. In some categories of infants, such as those born before 28 completed weeks of gestation and infants with antenatally-diagnosed major congenital anomalies, antenatal planning is mandated by national guidelines to ensure transfer of women to a tertiary centre for delivery (prenatal or in utero transfers) [2]. For these high-risk infants, birth in a hospital with a tertiary unit is associated with better neonatal outcomes such as lower odds of severe brain injury and higher odds of survival without severe brain injury compared to infants born in a hospital without a tertiary NICU and postnatally transferred to a tertiary NICU after stabilisation [5]. Birth in a tertiary unit is also associated with better outcomes in late or moderately born preterm infants [6].

Despite the aforementioned better outcomes associated with birth in a hospital with a tertiary neonatal unit, birth is a natural process and most babies are born healthy requiring little or no medical intervention. Neonatal care cannot be confined only to geographical areas with tertiary neonatal units and all pregnant women have a discussion around birth setting within their local area unless there are indications to deliver in a hospital with tertiary neonatal services. It is also important to note that infants at or close to term can be unexpectedly

Confidential: For Review Only

unwell and that premature birth cannot always be predicted resulting in unexpected births of sick and preterm infants in hospitals without tertiary neonatal services [7].

Better outcomes in tertiary NICUs result from the presence of medical and surgical subspecialties, increased funding, enhanced training and sophisticated equipment. Perhaps, however, the most important factor is the highly skilled staff who work in these units: the neonatal nurses, doctors and allied health professionals such as dieticians, physiotherapists and speech and language therapists. As not all infants can be delivered in hospitals with tertiary units, neonatal outcomes could, thus, be improved by allocating enhanced resources to non-tertiary neonatal units and cross-site working of skilled neonatal professionals that work primarily in a tertiary setting and can be partly deployed to non-tertiary units.

King's College Hospital NHS Foundation Trust (KCH) has a busy surgical and medical NICU and is located in South East London. KCH acquired in 2013 a level one neonatal unit at the Princess Royal University Hospital (PRUH) and gradually implemented cross-site working of doctors, nurses and allied health professionals. We hypothesised that the implementation of cross-site working between a level three and a level one neonatal unit would be associated with improved short-term neonatal outcomes. Our aim was to test this hypothesis by comparing outcomes before and after the implementation of this model.

MATERIALS AND METHODS

Subjects and study design

A retrospective cohort study of all admissions to the Neonatal Unit at the PRUH between 1st January 2010 and 1st January 2022 was undertaken. The PRUH is located at Farnborough Common, Orpington, South East London, United Kingdom and is responsible for approximately 4,000 deliveries and 350-400 neonatal admissions per year [8]. The hospital has a level one neonatal unit with 10 cots. Level one units in the UK look after infants requiring special care and are suitable for deliveries at 32 weeks of gestation and above that

Confidential: For Review Only

are considered low risk and deliveries at 30-32 weeks of gestation subject to risk assessment. Infants requiring intensive care (for example invasive ventilation) or high dependency care (for example parenteral nutrition or short term invasive ventilation) are transferred to a higher level unit [1, 3]. Only infants who were born in the PRUH were included in this study; infants who were born in another hospital or whose main care was provided in another hospital and were transferred to the PRUH for continuation of care or pre-discharge, were not included as they were not thought to be representative of the care provided in the PRUH. If an infant had multiple admissions to the unit following referral from a local hospital or repatriation following specialist care, only the first episode was included in the analysis.

The level three unit of this study, was the tertiary neonatal intensive care centre of KCH NHS Trust, London, UK. KCH has a tertiary medical and surgical neonatal unit with 36 cots, with approximately 5,000 deliveries and 700 neonatal admissions per year. It serves a diverse community of over 1,000,000 in South East London and is part of the London Neonatal Operational Delivery Network. Level 3 units in the United Kingdom are suitable for deliveries of infants at 27 weeks and below as well as infants that require high dependency and special care [2, 4].

The study was registered with the Clinical Governance Department of King's College Hospital NHS Foundation Trust. The Health Research Authority Toolkit of the National Health System, United Kingdom confirmed that the study was not considered as research and hence would not need regulatory approval by a research ethics committee.

Confidential: For Review Only

Data collected from the medical and nursing notes

Data were extracted from the BadgerNet Neonatal Electronic Patient Database (Clevermed, Edinburgh, UK). Mortality was defined as death before discharge from neonatal care [9]. The following data were collected:

Labour and delivery:

Administration of any antenatal steroids (yes/no), cord arterial pH, cord arterial pH <7.10 [10], gestational age (weeks), birth weight (kg), sex (male/female), Apgar score at 10 minutes, time of admission, admission temperature (^{o}C), admission blood glucose (mmol/L).

Neonatal care:

Time to first consultation by a senior member of staff (Consultant, nurse in charge or senior trainee - in hours), mechanical ventilation (yes/no), pneumothorax diagnosed on chest radiography (yes/no), whole body hypothermia (cooling) for hypoxic ischemic encephalopathy (yes/no), discharge home on supplemental oxygen (yes/no), postmenstrual age at discharge (weeks), total length of stay (days).

The birthweight z-score was calculated using the UK-WHO preterm reference chart [11] and the Microsoft Excel add-in LMSgrowth (V.2.77; www.healthforchildren.co.uk). Hypothermia was defined as an admission temperature of less than 36.5 ^{o}C [12]. Hypoglycaemia was defined as a blood glucose concentration of less than 2.6 mmol/L [13].

Periods of implementation of cross-site working

Elements of the intervention

A comprehensive, gradual quality improvement programme was implemented that included up-skilling of the staff with cross-site education, training in newborn life support, simulation scenarios, regular multi-professional case review meetings and increased levels of staffing. Individual cases were regularly discussed for a second opinion with the senior neonatal Consultant on service at KCH which formed a separate rota of neonatal Consultants to the one at the PRUH. The neonatal team at KCH offered support by discussing individual cases and facilitated locating tertiary cots and transfer for tertiary care when required. Although not

Confidential: For Review Only

covered in detail in this report, a similar programme was undertaken by the maternity team aiming to improve maternity outcomes across both sites.

Pre cross-site period

The PRUH was acquired by KCH in October 2013. The first dedicated neonatal Consultant was in post in September 2014 and before this the paediatric Consultants covering the PRUH unit covered the neonatal unit as well as the paediatric wards and paediatric Accident and Emergency. The clinical cover for the neonatal unit in the pre-cross site period consisted of a Consultant covering both the paediatric and neonatal side and a middle grade doctor (Registrar) covering both the paediatric and neonatal sides of the service. For purposes of data entry consistency and to reflect current neonatal practice, the start point of the pre cross-site period was set in 2010. The pre-cross site period, thus, consisted of the chronological years 2010-2014 (five years).

Washout period

The cross-site working model was partly introduced in 2015 with complete implementation in late 2018 when a total of six cross-site neonatal Consultants were in post and formed a separate cross-site rota. In the years 2015-2018 a number of other changes were gradually introduced. Three fixed-term (one year) neonatal consultants covering only the PRUH neonatal unit were appointed in 2016 and 2017. An additional dedicated neonatal tier of middle grade doctors (registrars) who covered only the neonatal unit (and not the paediatric ward and the emergency department) was introduced in 2017. Four-week nursing placements of the PRUH nurses to KCH were introduced in 2017 and reciprocal nursing placements of KCH nurses to the PRUH were introduced in 2018. Cross-site clinical guidelines were also gradually introduced and implemented in 2017 and 2018. The washout period between 2015 and 2018 was deemed essential as the aforementioned changes were introduced during this period and not all Consultant posts were consistently filled. The washout period thus corresponded to the chronological years of 2015-2018 (four years).

Confidential: For Review Only

Cross-site period

A complete rota of cross-site neonatal Consultants that were working across both KCH and the PRUH was fully implemented by 2019. A cross-site Neonatal Lead Nurse for both sites was appointed in 2019. Nursing placements of the PRUH nurses to KCH were achieved with 80% of senior nurses undertaking a placement/rotation by 2019. The cross-site period thus, consisted of the chronological years 2019-2021 (three years). The year 2022 was not included as the data collection for this projected started in April 2022. The total number of beds did not change during the study period.

Statistical analysis

Continuous data were tested for normality with visual inspection of their distribution curves and the Kolmogorov-Smirnov test and were found to be not normally distributed. Data were thus presented as median (interquartile range). The Mann Whitney U test was used for comparisons of variables before and after the implementation of the cross-site working model. Binary variables were compared between the two periods with the chi square test. The temporal evolution of the length of stay in days, the time to first consultation by a senior member of the team in hours and the admission temperature in ^{o}C were presented in boxplots with each year presented as a separate boxplot. The length of stay was divided into quintiles and the quintile corresponding to the longer duration of stay was used as the dependent variable in a binary multivariable regression model with gestational age, birth weight z-score, admission temperature, arterial cord pH and admission year as covariates. This multivariable regression model was constructed to describe the relative contributions of the selected covariates to a longer duration of stay. The continuous parameter "admission year" was used instead of the binary "cross site period" to minimise type I and type II error by dichotomising a continuous variable.

Statistical analysis was performed using SPSS V.26.0 software.

Confidential: For Review Only

RESULTS

A total of 4,418 infants were admitted to the neonatal unit at the PRUH during the study period. A total of 303 infants were repatriated to the PRUH having been born in another hospital and were thus excluded from the analysis. In the pre cross-site period, 1,299 infants and in the cross-site period 1,052 infants were born and admitted in the neonatal unit of the PRUH. In the washout period a total of 1,765 infants were born and admitted in the neonatal unit (data not presented).

The characteristics and outcomes of the included infants in the two study periods are presented in table 1. The percentage of infants delivering at the PRUH at a gestation below 32 weeks was higher in the pre cross-site period compared to the cross site period ($p<0.001$). The percentage of infants with an Apgar score of less than 8 at 10 minutes of life, was higher in the pre cross-site period compared to the cross site period ($p=0.001$). More infants were admitted with hypothermia and an admission temperature of less than 36°C in the pre-cross site period compared to the cross site period ($p<0.001$). Furthermore, the median duration of time to the first consultation by a member of the admitting team was higher in the pre cross-site period compared to the cross-site period ($p<0.001$) with more parents receiving the first consultation/update after four hours from the time of admission compared to the cross-site period ($p<0.001$). The median length of stay was higher in the pre cross-site period compared to the cross-site period ($p<0.001$). The incidence of an arterial pH of less than 7.1 was higher in the infants in whom cord gases were performed in the pre cross-site period compared to the cross-site period ($p<0.001$). There were no significant differences in the administration of antenatal steroids, admission hypoglycaemia and therapeutic cooling between the two study periods (table 1). The evolution of the length of stay, time to first consultation and admission temperature over the years of the study period is presented in figure 1.

Confidential: For Review Only

Table 1: Characteristics and outcomes of the admitted infants in the two study periods.

Median (IQR) or *N* (%)

	Pre cross-site *N*=1,299	Cross-site *N*=1,052	p value
Antenatal steroids	451 (34.7)	310 (29.5)	0.127
Cord arterial pH	7.25 (7.12 – 7.32)	7.27 (7.19 – 7.32)	0.003
Cord arterial pH<7.1	97 of 516 (18.8)	57 of 592 (9.6)	<0.001
Gestational age (weeks)	37.1 (34.1 – 39.9)	38.8 (35.9– 40.3)	<0.001
Gestation < 32 weeks	116 (8.9)	38 (3.6)	<0.001
Birth weight (kg)	2.76 (2.01 – 3.45)	3.14 (2.50 – 3.61)	<0.001
Birth weight z score	-0.15 (-0.83 – 0.53)	-0.06 (-0.77 – 0.60)	0.019
Male sex	773 (59.5)	589 (56.0)	0.217
Apgar at 10 minutes <8	81 (6.2)	36 (3.4)	0.001
Admission hypothermia	346 (26.6)	180 (17.1)	<0.001
Admission temperature<36ºC	160 (12.3)	39 (3.7)	<0.001
Admission hypoglycaemia	327 (25.1)	245 (23.3)	0.193
Time to first consultation (h)	1.0 (0.5 – 2.6)	0.5 (0.2 – 1.3)	<0.001
Time to first consultation >4h	134 (10.3)	57 (5.4)	<0.001
Pneumothorax	18 (1.4)	19 (1.8)	0.528
Cooling	17 (1.3)	14 (1.3)	0.961
Home oxygen	129 (9.9)	146 (13.8)	<0.001
Mortality	12 (0.9)	1 (0.1)	<0.001
Length of stay (days)	4 (2 – 11)	2 (1 – 4)	<.0.001
Postmenstrual age at discharge (wks)	38.1 (36.0 – 40.2)	39.1 (36.5 – 40.4)	<0.001
Weight at discharge	2.66 (2.04 – 3.39)	3.08 (2.42 – 3.58)	<0.001

Comparisons by Mann-Whitney U or Chi square test as appropriate.

Confidential: For Review Only

Following binary regression analysis, the highest quintile of the length of stay (10-89 days) was independently associated with the admission year (Odds ratio: 0.90, 95% CI: 0.86 -0.93, adjusted p<0.001), gestational age (Odds ratio: 0.72, 95%CI: 0.69 – 0.75), adjusted p<0.001), birth weight z-score (Odds ratio: 0.71, 95% CI: 0.63 – 0.81, adjusted p<0.001), admission temperature (Odds ratio: 0.18, 95% CI: 1.33 – 2.31, adjusted p<0.001) and cord pH (Odds ratio: 17.75, 95% CI: 5.09 – 61.81, adjusted p<0.001).

DISCUSSION

We have demonstrated that cross-site working between a level three and a level one neonatal unit was associated with improved short-term neonatal outcomes including less admission hypothermia, shorter duration of stay and more timely first consultation by senior members of the team.

Our results highlight a successful method in which a level-one unit can maintain and improve quality standards by working together with a tertiary level. This has implications within networks of neonatal care, as such models could be adopted more widely to improve the care of infants born in hospitals without tertiary neonatal facilities. Of note, the results of our study relate to a unit with a workload which is higher compared to other level one units in London in the same study periods. A recent survey of neonatal units reported live births ranging from two to three thousands per year in other level one units in London [14]. Our method thus could be feasible in the lower-volume level one units.

We could not compare our study results with studies reporting on interventions to improve neonatal outcomes on level one units, as to the best of our knowledge there are no other such studies in the literature. Cross-site working between a level three and a level one unit is not the only potential model of care that could theoretically improve neonatal outcomes in level one units. Other described models include the development of the roles of the enhanced or advanced neonatal nurse practitioners, physician associates or associate

https://mc.manuscriptcentral.com/bmjpo

Confidential: For Review Only

specialists [14]. These, however, are mostly recent developments with a paucity of published evidence on the impact of these models on neonatal outcomes. A number of other factors can affect neonatal outcomes such as the impact of culture and morale on the workforce, investment in clinical supervision and training, implementation of quality improvement projects and integrated family development care. We could not capture these outcomes, as they are not quantified and documented in the neonatal data entry software [14].

Some of the improved outcomes that we have reported following the introduction of the cross-site working model, might also be explained by improved maternity care which might be the product of cross-site working in the maternity services. Such improved outcomes include a smaller percentage of infants born with a low cord pH and a lower number of infants born at less than 32 weeks of gestation, signalling more efficient in utero transfers of women at risk. The consistent presence of neonatal Consultants who did not have commitments to other paediatric services facilitated better and more frequent communication and interaction between the neonatal team and the obstetric and midwifery teams. Furthermore, improved admission temperature might also be related to better maternity care in the delivery suite, obstetric theatres and postnatal wards. The non-significant increase in the administration of antenatal steroids might be explained by the smaller percentage of infants that delivered prematurely in the PRUH and would thus qualify for this intervention [15]. The smaller percentage of infants that had a lower Apgar score at 10 minutes might be related to improved resuscitation at birth, but could also be partly explained by the smaller number of infants that were born with a lower cord arterial pH. We recognise, thus, that maternity improvement clearly played a major part in our improved neonatal outcomes. The majority, though, of the reported outcomes in our study are direct quality indicators of neonatal care and could be partly attributed to improved multi-professional neonatal care [1]. We should also note that the smaller percentage of infants that delivered below 32 weeks of gestation in the cross-site period might have contributed to some improved outcomes that

Confidential: For Review Only

are more relevant to preterm infants such as admission hypothermia and a decreased length of stay.

Our study has significant financial implications. Neonatal services are costly as they require expensive equipment, consumables and employment of a skilled multi-professional workforce which operates 24 hours a day. Neonatal units have been reported to have an incomplete understanding of the costs of running the service [16]. We did not perform a separate financial analysis, as all care days were at the same level and cost. We reported, however, that the median length of stay was halved, which corresponds to a cost reduction of a similar magnitude. It follows that if this intervention was implemented on a wider scale, it could be associated with significant cost reductions. Daily charges for neonatal care vary across different Trusts, with little consensus on the basis on which these charges are determined, but an average cost of special care per day is approximately 500 GBP [17]. At a single level one unit, thus, with 400 admissions per year the cost saving would be approximately 100,000 GBP per year. We do not present in this paper a full financial analysis of the intervention but these numbers should be taken in the context of the relevant staffing and equipment expenditure. Irrespective of the financial aspects, it is important to remember that decreased length of stay and earlier discharge by senior clinicians coupled with appropriate outpatient follow-up arrangements promote normal family life in a home environment with enhanced quality of sleep, less exposure to infection risks [18] and improved neurodevelopment [19].

Interestingly, the introduction of a cross-site model was not associated with a reduction of the total number of admissions per year. This might be explained by the fact that newborn infants constitute an already high-risk patient population. According to the World Health Organisation in 2019, 47% of all deaths in under five year olds occurred in the newborn period [20]. Previous neonatal studies have focused on improving outcomes of premature infants and more so the extremely premature ones. It is interesting, however, to note that the vast majority of births are term or late preterm and neonatal admissions are commonly

Confidential: For Review Only

infants who require a brief episode of care. Our study is thus relevant to a wider population than the results of the specialised studies of extreme prematurity and more applicable to the wider public.

Our study has strengths and some limitations. We used a large population of infants that were cared for on the same unit minimising potential differences that relate to different clinical practices or standards of care between units. The applicability of our study lies in that the improved short-term neonatal outcomes following the introduction of cross-site working, if confirmed by larger and more diverse-population studies, could strengthen the argument of implementing a cross-site neonatal model of care. We acknowledge as a limitation that it is impossible to completely separate the relative contribution of cross-site working to improved outcomes from the overall global tendency for better outcomes in neonatal care. For example, there has been an international drive to recognise and prevent neonatal hypothermia following the recognition of the detrimental effects of this complication on neonatal outcomes [21]. The International Liaison Committee on Resuscitation recommended in a consensus statement in 2015 that the admission temperature of newborn infants should be maintained between 36.5 and 37.5 ^{o}C after birth, during stabilisation and admission, as temperature was a strong predictor of mortality and morbidity [22]. Other outcomes though, such as earlier consultation and discharge and timely in utero transfer, can be predominantly attributed to the implementation of novel workforce models such as cross-site working. Our study cannot establish a clear causal link between the introduction of cross-site working and improved neonatal outcomes as it is retrospective and spans over twelve years. Nevertheless, it is important to share with the neonatal community our experience of this intervention which could have significant policy implications.

In conclusion, cross-site working between a level three and a level one neonatal unit was associated with improved short-term neonatal outcomes relevant to level one neonatal care, such as a reduction in admission hypothermia, shorter length of stay and more timely first consultation by senior members of the team.

Confidential: For Review Only

Legend to figures

Figure 1: Evolution of the length of stay (a), time to first consultation (b) and admission temperature (c) over the study period. The horizontal lines in the boxes represent the lower quartile, median and upper quartile values of the presented parameters and the whiskers the minimum and maximum values. The whiskers do not include the outliers, defined as values more than one and a half box lengths from the median which are presented as open circles. The whiskers do not include the extreme outliers, defined as values more than three box lengths from the median which are presented as asterisks.

Confidential: For Review Only

Funding statement: This project was partially supported by King's College London 2018 Medical Research Council Confidence in Concept Award through the King's Health Partners' Research and Development Challenge Fund.

Competing interests statement: The authors have no competing interests to declare.

Author's contribution: TD conceived the study, contributed to data analysis and interpretation and wrote the first version of the manuscript. LS collected the data and contributed to data analysis and writing the manuscript. AH led the implementation of the intervention, contributed to study design and critically revised the manuscript. ES lead the implementation of the intervention and critically revised the manuscript. LL contributed to the study design and critically revised the manuscript. LP contributed to the study design and critically revised the manuscript. VWM contributed to the study design and critically revised the manuscript. RB led the implementation of the intervention, contributed to study design and critically revised the manuscript. AG contributed to project supervision, funding acquisition and extensively revised and edited the manuscript. All authors have read and agreed to the published version of the manuscript.

Data sharing statement: Data is available to share upon request.

Confidential: For Review Only

References

1. NHS. Toolkit for High Quality Neonatal Services. London, UK2009.

2. Improvement NEaN. Implementing the Recommendations of the Neonatal Critical Care Transformation Review. In: Improvement NEaN, editor. 2019.

3. BAPM. Service standards for hospitals providing neonatal care. 3rd Edition. 2010.

4. Luu TM, Rehman Mian MO, Nuyt AM. Long-Term Impact of Preterm Birth: Neurodevelopmental and Physical Health Outcomes. Clinics in perinatology. 2017 Jun;44(2):305-14. PubMed PMID: 28477662. Epub 2017/05/10.

5. Helenius K, Longford N, Lehtonen L, Modi N, Gale C, Neonatal Data Analysis U, et al. Association of early postnatal transfer and birth outside a tertiary hospital with mortality and severe brain injury in extremely preterm infants: observational cohort study with propensity score matching. Bmj. 2019 Oct 16;367:l5678. PubMed PMID: 31619384. Pubmed Central PMCID: PMC6812621 www.icmje.org/coi_disclosure.pdf and declare: no support from any organisation for the submitted work; no financial relationships with any organisations that might have an interest in the submitted work in the previous three years; no other relationships or activities that could appear to have influenced the submitted work. Epub 2019/10/18.

6. Boyle EM, Johnson S, Manktelow B, Seaton SE, Draper ES, Smith LK, et al. Neonatal outcomes and delivery of care for infants born late preterm or moderately preterm: a prospective population-based study. Archives of disease in childhood Fetal and neonatal edition. 2015 Nov;100(6):F479-85. PubMed PMID: 25834169. Pubmed Central PMCID: PMC4680176. Epub 2015/04/03.

7. Suff N, Story L, Shennan A. The prediction of preterm delivery: What is new? Seminars in fetal & neonatal medicine. 2019 Feb;24(1):27-32. PubMed PMID: 30337215. Epub 2018/10/20.

Confidential: For Review Only

8. https://www.ons.gov.uk/aboutus/transparencyandgovernance/freedomofinformationfoi/hospitalstatistics: Office for National Statistics; [cited 2022 03/05].

9. Smith LK, Draper ES, Manktelow BN, Field DJ. Socioeconomic inequalities in survival and provision of neonatal care: population based study of very preterm infants. Bmj. 2009 Dec 1;339:b4702. PubMed PMID: 19952036. Pubmed Central PMCID: PMC2786957. Epub 2009/12/03.

10. Yeh P, Emary K, Impey L. The relationship between umbilical cord arterial pH and serious adverse neonatal outcome: analysis of 51,519 consecutive validated samples. BJOG. 2012 Jun;119(7):824-31. PubMed PMID: 22571747. Epub 2012/05/11.

11. Wright CM, Booth IW, Buckler JM, Cameron N, Cole TJ, Healy MJ, et al. Growth reference charts for use in the United Kingdom. Archives of disease in childhood. 2002 Jan;86(1):11-4. PubMed PMID: 11806873. Pubmed Central PMCID: PMC1719041. Epub 2002/01/25.

12. Laptook AR, Bell EF, Shankaran S, Boghossian NS, Wyckoff MH, Kandefer S, et al. Admission Temperature and Associated Mortality and Morbidity among Moderately and Extremely Preterm Infants. The Journal of pediatrics. 2018 Jan;192:53-9 e2. PubMed PMID: 29246358. Pubmed Central PMCID: PMC5808888. Epub 2017/12/17.

13. BAPM. Identification and Management of Neonatal Hypoglycaemia in the Full Term Infant. 2017.

14. Mitra T, Bramwells L. Exploring New Ways of Working in the Neonatal Unit. London: London Neonatal Operational Delivery Network; 2017.

15. Chawla S, Natarajan G, Shankaran S, Pappas A, Stoll BJ, Carlo WA, et al. Association of Neurodevelopmental Outcomes and Neonatal Morbidities of Extremely Premature Infants With Differential Exposure to Antenatal Steroids. JAMA pediatrics. 2016 Dec 1;170(12):1164-72. PubMed PMID: 27723868. Pubmed Central PMCID: PMC5294968. Epub 2016/10/11.

Confidential: For Review Only

16. Accounts HoCCoP. Caring for Vulnerable Babies: The reorganisation of neonatal services in England. London House of Commons; 2008.

17. Dassios T, Greenough A, Leontiadi S, Hickey A, Kametas NA. Admissions for hypoglycaemia after 35 weeks of gestation: perinatal predictors of cost of stay. The journal of maternal-fetal & neonatal medicine : the official journal of the European Association of Perinatal Medicine, the Federation of Asia and Oceania Perinatal Societies, the International Society of Perinatal Obstet. 2019 Feb;32(3):448-54. PubMed PMID: 28922987. Epub 2017/09/20.

18. Jones E, Stewart F, Taylor B, Davis PG, Brown SJ. Early postnatal discharge from hospital for healthy mothers and term infants. The Cochrane database of systematic reviews. 2021 Jun 8;6:CD002958. PubMed PMID: 34100558. Pubmed Central PMCID: PMC8185906. Epub 2021/06/09.

19. Losbar JL, Montjaux N, Ehlinger V, Germany L, Arnaud C, Tscherning C. Early discharge and hospital-assisted home care is associated with better neurodevelopmental outcome in preterm infants. Early human development. 2021 Oct;161:105451. PubMed PMID: 34507020. Epub 2021/09/11.

20. WHO. Newborns: improving survival and well-being: WHO 2020 [cited 2022 02/05]. Available from: https://www.who.int/news-room/fact-sheets/detail/newborns-reducing-mortality#:~:text=Causes,within%20the%20first%2024%20hours.

21. Lunze K, Bloom DE, Jamison DT, Hamer DH. The global burden of neonatal hypothermia: systematic review of a major challenge for newborn survival. BMC medicine. 2013 Jan 31;11:24. PubMed PMID: 23369256. Pubmed Central PMCID: PMC3606398. Epub 2013/02/02.

22. Perlman JM, Wyllie J, Kattwinkel J, Wyckoff MH, Aziz K, Guinsburg R, et al. Part 7: Neonatal Resuscitation: 2015 International Consensus on Cardiopulmonary Resuscitation and Emergency Cardiovascular Care Science With Treatment Recommendations. Circulation. 2015 Oct 20;132(16 Suppl 1):S204-41. PubMed PMID: 26472855. Epub 2015/10/17.

Confidential: For Review Only

Confidential: For Review Only

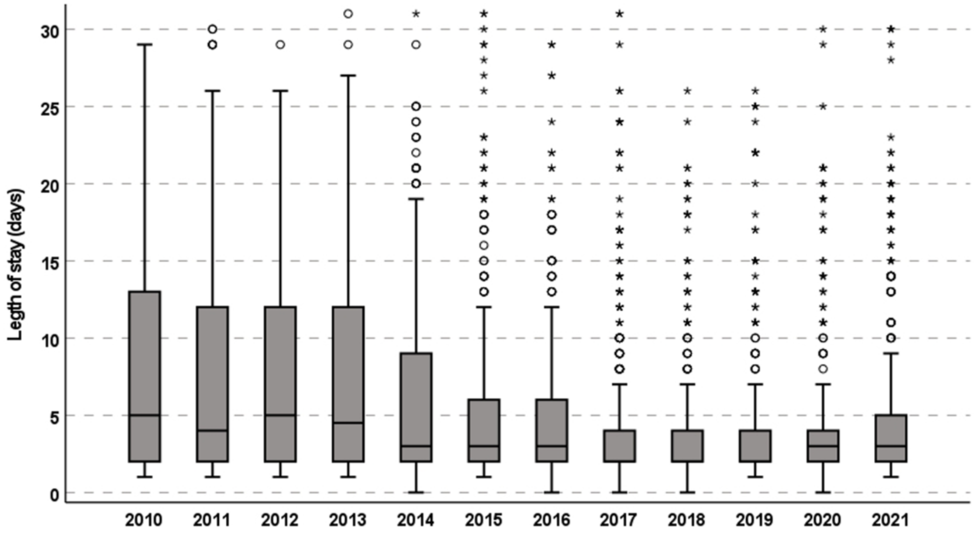

Figure 1: Evolution of the length of stay (a), time to first consultation (b) and admission temperature (c) over the study period. The horizontal lines in the boxes represent the lower quartile, median and upper quartile values of the presented parameters and the whiskers the minimum and maximum values. The whiskers do not include the outliers, defined as values more than one and a half box lengths from the median which are presented as open circles. The whiskers do not include the extreme outliers, defined as values more than three box lengths from the median which are presented as asterisks.

300x177mm (300 x 300 DPI)

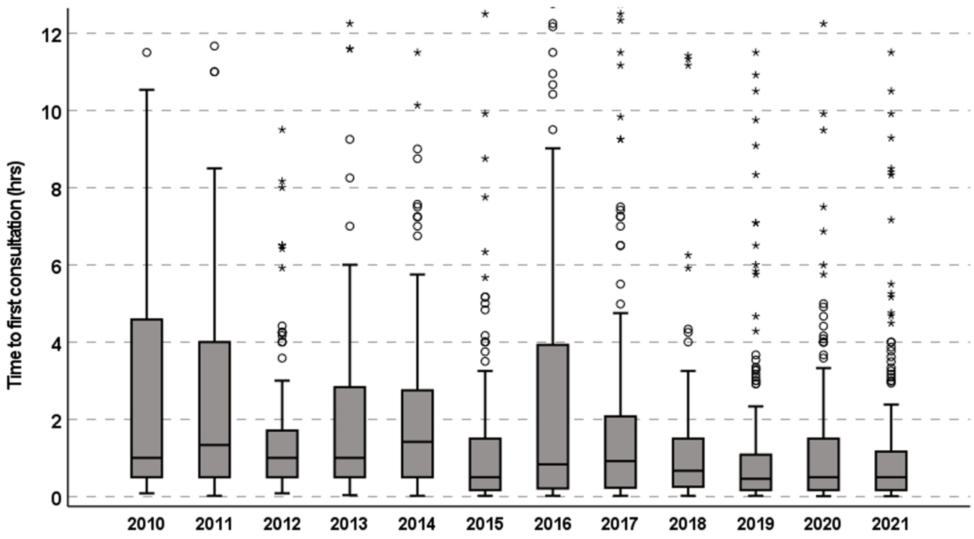

Figure 1: Evolution of the length of stay (a), time to first consultation (b) and admission temperature (c) over the study period. The horizontal lines in the boxes represent the lower quartile, median and upper quartile values of the presented parameters and the whiskers the minimum and maximum values. The whiskers do not include the outliers, defined as values more than one and a half box lengths from the median which are presented as open circles. The whiskers do not include the extreme outliers, defined as values more than three box lengths from the median which are presented as asterisks.

300x177mm (300 x 300 DPI)

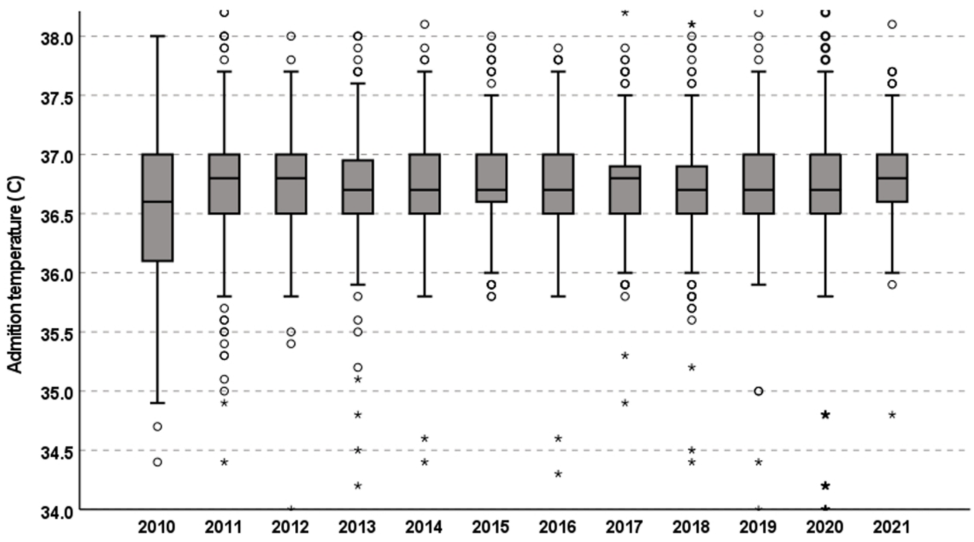

Figure 1: Evolution of the length of stay (a), time to first consultation (b) and admission temperature (c) over the study period. The horizontal lines in the boxes represent the lower quartile, median and upper quartile values of the presented parameters and the whiskers the minimum and maximum values. The whiskers do not include the outliers, defined as values more than one and a half box lengths from the median which are presented as open circles. The whiskers do not include the extreme outliers, defined as values more than three box lengths from the median which are presented as asterisks.

300x177mm (300 x 300 DPI)

Go straight to content.

Medical Research Council

NHS Health Research Authority

Is my study research?

To print your result with title and IRAS Project ID please enter your details below:

Title of your research:

Multi-professional cross-site working between a level one and a level three neonatal unit: Impact on clinica[illegible]
outcomes

Corresponding author:
Dr Theodore Dassios, PhD, FRCPCH
Reader in Neonatology, School of Life Course Sciences, King's College London,
Consultant Neonatologist, King's College Hospital NHS Foundation Trust,
Neonatal Unit, Golden Jubilee Wing, King's College Hospital,
Denmark Hill, SE5 9RS, London, UK

IRAS Project ID (if available): Not applicable

You selected:

- **'No'** - Are the participants in your study randomised to different groups?
- **'No'** - Does your study protocol demand changing treatment/ patient care from accepted standards for any of the patients involved?
- **'No'** - Are your findings going to be generalisable?

Your study would NOT be considered Research by the NHS.

You may still need other approvals.

Researchers requiring further advice (e.g. those not confident with the outcome of this tool) should contact their R&D office or sponsor in the first instance, or the HRA to discuss your study. If contacting the HRA for advice, do this by sending an outline of the project (maximum one page), summarising its purpose, methodology, type of participant and planned location as well as a copy of this results page and a summary of the aspects of the decision(s) that you need further advice on to the HRA Queries Line at Queries@hra.nhs.uk.

For more information please visit the Defining Research table.

Follow this link to start again.

Print This Page

NOTE: If using Internet Explorer please use browser print function.

About this tool Feedback Contact Glossary Accessibility

BMJ Paediatrics Open

Multi-professional cross-site working between a level one and a level three neonatal unit: a retrospective cohort study

Journal:	*BMJ Paediatrics Open*
Manuscript ID	bmjpo-2022-001581.R2
Article Type:	Original research
Date Submitted by the Author:	26-Aug-2022
Complete List of Authors:	Dassios, Theodore; King's College Hospital NHS Foundation Trust, Neonatal Intensive Care Unit; King's College London, Department of Women and Children’s Health, School of Life Course Sciences, Faculty of Life Sciences and Medicine<br>Selvadurai, Lucksini; King's College Hospital NHS Foundation Trust, Neonatal Intensive Care Unit<br>Hickey, Ann; King's College Hospital NHS Foundation Trust, Neonatal Intensive Care Unit<br>Sleight, Elizabeth; King's College Hospital NHS Foundation Trust, Neonatal Intensive Care Unit<br>Long, Lisa; King's College Hospital NHS Foundation Trust, Department of Obstetrics and Gynaecology<br>Penna, Leonie; King's College Hospital NHS Foundation Trust, Department of Obstetrics and Gynaecology<br>Wallen-Mitchell, Vivette; King's College Hospital NHS Foundation Trust, Neonatal Intensive Care Unit<br>Bhat, Ravindra; King's College Hospital NHS Foundation Trust, Neonatal Intensive Care Unit<br>Greenough, Anne; King's College London, Women and Children's Health, School of Life Course Sciences; NIHR Biomedical Research Centre at Guy's and Saint Thomas' NHS Foundation Trust and King's College
Keywords:	Neonatology

SCHOLARONE™
Manuscripts

I, the Submitting Author has the right to grant and does grant on behalf of all authors of the Work (as defined in the below author licence), an exclusive licence and/or a non-exclusive licence for contributions from authors who are: i) UK Crown employees; ii) where BMJ has agreed a CC-BY licence shall apply, and/or iii) in accordance with the terms applicable for US Federal Government officers or employees acting as part of their official duties; on a worldwide, perpetual, irrevocable, royalty-free basis to BMJ Publishing Group Ltd ("BMJ") its licensees and where the relevant Journal is co-owned by BMJ to the co-owners of the Journal, to publish the Work in this journal and any other BMJ products and to exploit all rights, as set out in our licence.

The Submitting Author accepts and understands that any supply made under these terms is made by BMJ to the Submitting Author unless you are acting as an employee on behalf of your employer or a postgraduate student of an affiliated institution which is paying any applicable article publishing charge ("APC") for Open Access articles. Where the Submitting Author wishes to make the Work available on an Open Access basis (and intends to pay the relevant APC), the terms of reuse of such Open Access shall be governed by a Creative Commons licence – details of these licences and which Creative Commons licence will apply to this Work are set out in our licence referred to above.

Other than as permitted in any relevant BMJ Author's Self Archiving Policies, I confirm this Work has not been accepted for publication elsewhere, is not being considered for publication elsewhere and does not duplicate material already published. I confirm all authors consent to publication of this Work and authorise the granting of this licence.

Confidential: For Review Only

Multi-professional cross-site working between a level one and a level three neonatal unit: a retrospective cohort study

Theodore Dassios[1,2], Lucksini Selvadurai[1], Ann Hickey[1], Elizabeth Sleight[1], Lisa Long[3], Leonie Penna[3], Vivette Wallen-Mitchell[1], Ravindra Bhat[1], Anne Greenough[2,4].

[1]Neonatal Intensive Care Centre, King's College Hospital NHS Foundation Trust, London, United Kingdom;

[2]Department of Women and Children's Health, School of Life Course Sciences, Faculty of Life Sciences and Medicine, King's College London, London, United Kingdom;

[3]Department of Obstetrics and Gynaecology, King's College Hospital NHS Foundation Trust, London, United Kingdom;

[4]NIHR Biomedical Centre at Guy's and St Thomas NHS Foundation Trust and King's College London, London, United Kingdom

Address for correspondence: Dr Theodore Dassios, Neonatal Intensive Care Centre, 4th Floor Golden Jubilee Wing, King's College Hospital, Denmark Hill, London, SE5 9RS, United Kingdom. Tel: +44 0203 299 4563; E-mail: theodore.dassios@kcl.ac.uk

Word count of manuscript: 3,877

Key words: cross-site working, neonatal outcomes, special care baby unit

Confidential: For Review Only

ABSTRACT

Objective: To assess the association of short-term neonatal outcomes with cross-site working of multiple healthcare professional teams between a level three and a level one neonatal unit.

Design: Retrospective cohort study.

Setting: A level one neonatal unit in London.

Patients: All infants admitted to the neonatal unit, between 2010 and 2021.

Interventions: The clinical service was rearranged in 2014 with the introduction of cross-site working between the level one unit and a level three unit of neonatal doctors, nurses and allied healthcare professionals.

Main outcome measures: Admission of infants with a temperature less than 36°C, length of stay and time to first consultation by a senior team member.

Results: A total of 4,418 infants were admitted during the study period. The percentage of infants delivered at a gestation below 32 weeks was higher in the pre cross-site period (8.9%) compared to the cross site period (3.6%, p<0.001). The percentage of infants with an Apgar score less than 8 at 10 minutes was higher in the pre cross-site period (6.2%) compared to the cross-site period (3.4%, p=0.001). More infants were admitted with a temperature less than 36°C in the pre-cross site period (12.3%) compared to the cross site period (3.7%, p<0.001). The median (IQR) duration of time to first consultation by a senior team member was higher in the pre cross-site period [1 (0.5 - 2.6) hours] compared to the cross-site period [0.5 (0.2 - 1.3) hours] (p<0.001). The median (IQR) length of stay was 4 (2-11) days in the pre cross-site period and decreased to 2 (1-4) days in the cross-site period (p<0.001).

Conclusions: Cross-site working was associated with lower rates of admission hypothermia, shorter duration of stay and earlier first senior consultation.

Confidential: For Review Only

Key messages

What is already known on this topic:

Birth of high risk infants in a hospital with a tertiary neonatal unit is associated with better short-term neonatal outcomes but neonatal care cannot be confined only to tertiary neonatal units.

What this study adds:

Cross-site working of neonatal doctors, nurses and allied healthcare professionals between a level three and a level one neonatal unit was associated with improved short-term neonatal outcomes, such as lower rates of admission hypothermia, shorter duration of stay and earlier first senior consultation.

How this study might affect research, practice or policy:

Cross site working models could be adopted more widely by the neonatal community to improve the care of infants born in hospitals without tertiary neonatal facilities.

Confidential: For Review Only

INTRODUCTION

In the United Kingdom, neonatal care is stratified in three progressively more complex levels: level one, which refers to special care baby units, level two or local neonatal units and the highly specialised level three (tertiary) or neonatal intensive care units (NICU) [1]. Neonatal care in England is currently delivered in 161 neonatal units out of which only 44 are tertiary centres with the remaining 70% being non-tertiary [2]. The allocation of a neonatal unit as tertiary or non-tertiary is based on clearly specified criteria relating to the level of clinical support that can be offered, staff seniority and training [1, 3]. The tertiary units care for the sickest of infants and provide intensive care such as prolonged invasive ventilation, multiple modes of cardiorespiratory support and subspecialty services.

Neonatal complications arising in the first hours and days of life have significant consequences which may influence lifelong health and quality of life [4]. In some categories of infants, such as those born before 28 completed weeks of gestation and infants with antenatally-diagnosed major congenital anomalies, antenatal planning is mandated by national guidelines to ensure transfer of women to a tertiary centre for delivery (prenatal or in utero transfers) [2]. For these high-risk infants, birth in a hospital with a tertiary unit is associated with better neonatal outcomes such as lower odds of severe brain injury and higher odds of survival without severe brain injury compared to infants born in a hospital without a tertiary NICU [5]. Birth in a tertiary unit is also associated with better outcomes in late or moderately born preterm infants [6].

Despite the aforementioned better outcomes associated with birth in a hospital with a tertiary neonatal unit, birth is a natural process and most babies are born healthy, requiring little or no medical intervention. Neonatal care cannot be confined only to geographical areas with tertiary neonatal units and all pregnant women have a discussion around birth setting within their local area unless there are indications to deliver in a hospital with tertiary neonatal services. It is also important to note that infants at or close to term can be unexpectedly

Confidential: For Review Only

unwell and that premature birth cannot always be predicted resulting in unexpected births of sick and preterm infants in hospitals without tertiary neonatal services [7].

Better outcomes in tertiary NICUs result from the presence of medical and surgical subspecialties, increased funding, enhanced training and sophisticated equipment. Perhaps, however, the most important factor is the highly skilled staff who work in these units: the neonatal nurses, doctors and allied health professionals such as dieticians, physiotherapists and speech and language therapists. As not all infants can be delivered in hospitals with tertiary units, neonatal outcomes could, thus, be improved by allocating enhanced resources to non-tertiary neonatal units and cross-site working of skilled neonatal professionals that work primarily in a tertiary setting and can be partly deployed to non-tertiary units.

King's College Hospital NHS Foundation Trust (KCH) has a busy surgical and medical NICU and is located in South East London. KCH acquired in 2013 a level one neonatal unit at the Princess Royal University Hospital (PRUH) and gradually implemented cross-site working of doctors, nurses and allied health professionals. We hypothesised that the implementation of cross-site working between a level three and a level one neonatal unit would be associated with improved short-term neonatal outcomes. Our aim was to test this hypothesis by comparing outcomes before and after the implementation of this model.

Confidential: For Review Only

MATERIALS AND METHODS

Subjects and study design

A retrospective cohort study of all admissions to the Neonatal Unit at the PRUH between 1st January 2010 and 1st January 2022 was undertaken. The PRUH is located at Farnborough Common, Orpington, South East London, United Kingdom and is responsible for approximately 4,000 deliveries and 350-400 neonatal admissions per year [8]. The hospital has a level one neonatal unit with 10 cots. Level one units in the UK look after infants requiring special care and are suitable for deliveries at 32 weeks of gestation and above that

are considered low risk and deliveries at 30-32 weeks of gestation subject to risk assessment. Infants requiring intensive care (for example invasive ventilation) or high dependency care (for example parenteral nutrition or short term invasive ventilation) are transferred to a higher level unit [1, 3]. Only infants who were born in the PRUH were included in this study; infants who were born in another hospital or whose main care was provided in another hospital and were transferred to the PRUH for continuation of care or pre-discharge, were not included as they were not thought to be representative of the care provided in the PRUH. If an infant had multiple admissions to the unit following referral from a local hospital or repatriation following specialist care, only the first episode was included in the analysis.

The level three unit of this study, was the tertiary neonatal intensive care centre of KCH NHS Trust, London, UK. KCH has a tertiary medical and surgical neonatal unit with 36 cots, with approximately 5,000 deliveries and 700 neonatal admissions per year. It serves a diverse community of over 1,000,000 in South East London and is part of the London Neonatal Operational Delivery Network. Level 3 units in the United Kingdom are suitable for deliveries of infants at 27 weeks and below as well as infants that require high dependency and special care [2, 4].

The study was registered with the Clinical Governance Department of King’s College Hospital NHS Foundation Trust. The Health Research Authority Toolkit of the National Health System, United Kingdom confirmed that the study was not considered as research and hence would not need regulatory approval by a research ethics committee.

Data collected from the medical and nursing notes

Data were extracted from the BadgerNet Neonatal Electronic Patient Database (Clevermed, Edinburgh, UK). Mortality was defined as death before discharge from neonatal care [9]. The following data were collected:

https://mc.manuscriptcentral.com/bmjpo

Confidential: For Review Only

Labour and delivery:

Administration of any antenatal steroids (yes/no), cord arterial pH, cord arterial pH <7.10 [10], gestational age (weeks), birth weight (kg), sex (male/female), Apgar score at 10 minutes, time of admission, admission temperature (^{o}C), admission blood glucose (mmol/L).

Neonatal care:

Time to first consultation by a senior member of staff (Consultant, nurse in charge or senior trainee - in hours), mechanical ventilation (yes/no), pneumothorax diagnosed on chest radiography (yes/no), whole body hypothermia (cooling) for hypoxic ischemic encephalopathy (yes/no), discharge home on supplemental oxygen (yes/no), postmenstrual age at discharge (weeks), total length of stay (days).

The birthweight z-score was calculated using the UK-WHO preterm reference chart [11] and the Microsoft Excel add-in LMSgrowth (V.2.77; www.healthforchildren.co.uk). Hypothermia was defined as an admission temperature of less than 36.5 ^{o}C [12]. Hypoglycaemia was defined as a blood glucose concentration of less than 2.6 mmol/L [13].

Periods of implementation of cross-site working

Elements of the intervention

A comprehensive, gradual quality improvement programme was implemented that included up-skilling of the staff with cross-site education, training in newborn life support, simulation scenarios, regular multi-professional case review meetings and increased levels of staffing. Individual cases were regularly discussed for a second opinion with the senior neonatal Consultant on service at KCH which formed a separate rota of neonatal Consultants to the one at the PRUH. The neonatal team at KCH offered support by discussing individual cases and facilitated locating tertiary cots and transfer for tertiary care when required. Although not

Confidential: For Review Only

covered in detail in this report, a similar programme was undertaken by the maternity team aiming to improve maternity outcomes across both sites.

Pre cross-site period

The PRUH was acquired by KCH in October 2013. The first dedicated neonatal Consultant was in post in September 2014 and before this the paediatric Consultants covering the PRUH unit covered the neonatal unit as well as the paediatric wards and paediatric Accident and Emergency. The clinical cover for the neonatal unit in the pre-cross site period consisted of a Consultant covering both the paediatric and neonatal side and a middle grade doctor (Registrar) covering both the paediatric and neonatal sides of the service. For purposes of data entry consistency and to reflect current neonatal practice, the start point of the pre cross-site period was set in 2010. The pre-cross site period, thus, consisted of the chronological years 2010-2014 (five years).

Washout period

The cross-site working model was partly introduced in 2015 with complete implementation in late 2018 when a total of six cross-site neonatal Consultants were in post and formed a separate cross-site rota. In the years 2015-2018 a number of other changes were gradually introduced. Three fixed-term (one year) neonatal consultants covering only the PRUH neonatal unit were appointed in 2016 and 2017. An additional dedicated neonatal tier of middle grade doctors (registrars) who covered only the neonatal unit (and not the paediatric ward and the emergency department) was introduced in 2017. Four-week nursing placements of the PRUH nurses to KCH were introduced in 2017 and reciprocal nursing placements of KCH nurses to the PRUH were introduced in 2018. Cross-site clinical guidelines were also gradually introduced and implemented in 2017 and 2018. The washout period between 2015 and 2018 was deemed essential as the aforementioned changes were introduced during this period and not all Consultant posts were consistently filled. The washout period thus corresponded to the chronological years of 2015-2018 (four years).

Confidential: For Review Only

Cross-site period

A complete rota of cross-site neonatal Consultants that were working across both KCH and the PRUH was fully implemented by 2019. A cross-site Neonatal Lead Nurse for both sites was appointed in 2019. Nursing placements of the PRUH nurses to KCH were achieved with 80% of senior nurses undertaking a placement/rotation by 2019. The cross-site period thus, consisted of the chronological years 2019-2021 (three years). The year 2022 was not included as the data collection for this project started in April 2022. The total number of beds did not change during the study period.

Statistical analysis

Continuous data were tested for normality with visual inspection of their distribution curves and the Kolmogorov-Smirnov test and were found to be not normally distributed. Data were thus presented as median (interquartile range). The Mann Whitney U test was used for comparisons of variables before and after the implementation of the cross-site working model. Binary variables were compared between the two periods with the chi square test. The temporal evolution of the length of stay in days, the time to first consultation by a senior member of the team in hours and the admission temperature in ^{o}C were presented in boxplots with each year presented as a separate boxplot. The length of stay was divided into quintiles and the quintile corresponding to the longer duration of stay was used as the dependent variable in a binary multivariable regression model with gestational age, birth weight z-score, admission temperature, arterial cord pH and admission year as covariates. This multivariable regression model was constructed to describe the relative contributions of the selected covariates to a longer duration of stay. The continuous parameter "admission year" was used instead of the binary "cross site period" to minimise type I and type II error by dichotomising a continuous variable. Multi-collinearity among the independent variables in the regression analysis was assessed by examination of a correlation matrix for the independent variables. Cox proportional hazards analysis with length of stay as the outcome

Confidential: For Review Only

variable and the year of admission and gestational age as covariates, was used to examine the effect of the admission year on the length of stay.

Statistical analysis was performed using SPSS V.26.0 software.

RESULTS

A total of 4,418 infants were admitted to the neonatal unit at the PRUH during the study period. A total of 303 infants were repatriated to the PRUH having been born in another hospital and were thus excluded from the analysis. In the pre cross-site period, 1,299 infants and in the cross-site period 1,052 infants were born and admitted in the neonatal unit of the PRUH. In the washout period a total of 1,765 infants were born and admitted in the neonatal unit (data not presented).

The characteristics and outcomes of the included infants in the two study periods are presented in table 1. The percentage of infants delivering at the PRUH at a gestation below 32 weeks was higher in the pre cross-site period compared to the cross site period ($p<0.001$). The percentage of infants with an Apgar score of less than 8 at 10 minutes of life, was higher in the pre cross-site period compared to the cross site period ($p=0.001$). More infants were admitted with hypothermia and an admission temperature of less than 36°C in the pre-cross site period compared to the cross site period ($p<0.001$). Furthermore, the median duration of time to the first consultation by a member of the admitting team was higher in the pre cross-site period compared to the cross-site period ($p<0.001$) with more parents receiving the first consultation/update after four hours from the time of admission compared to the cross-site period ($p<0.001$). The median length of stay was higher in the pre cross-site period compared to the cross-site period ($p<0.001$). The incidence of an arterial pH of less than 7.1 was higher in the infants in whom cord gases were performed in the pre cross-site period compared to the cross-site period ($p<0.001$). There were no significant differences in the administration of antenatal steroids, admission hypoglycaemia

Confidential: For Review Only

and therapeutic cooling between the two study periods (table 1). The evolution of the length of stay, time to first consultation and admission temperature over the years of the study period is presented in figure 1.

Confidential: For Review Only

Table 1: Characteristics and outcomes of the admitted infants in the two study periods.

Median (IQR) or *N* (%)

	Pre cross-site *N*=1,299	Cross-site *N*=1,052	p value
Antenatal steroids	451 (34.7)	310 (29.5)	0.127
Cord arterial pH	7.25 (7.12 – 7.32)	7.27 (7.19 – 7.32)	0.003
Cord arterial pH<7.1	97 of 516 (18.8)	57 of 592 (9.6)	<0.001
Gestational age (weeks)	37.1 (34.1 – 39.9)	38.8 (35.9– 40.3)	<0.001
Gestation < 32 weeks	116 (8.9)	38 (3.6)	<0.001
Birth weight (kg)	2.76 (2.01 – 3.45)	3.14 (2.50 – 3.61)	<0.001
Birth weight z score	-0.15 (-0.83 – 0.53)	-0.06 (-0.77 – 0.60)	0.019
Male sex	773 (59.5)	589 (56.0)	0.217
Apgar at 10 minutes <8	81 (6.2)	36 (3.4)	0.001
Admission hypothermia	346 (26.6)	180 (17.1)	<0.001
Admission temperature<36°C	160 (12.3)	39 (3.7)	<0.001
Admission hypoglycaemia	327 (25.1)	245 (23.3)	0.193
Time to first consultation (h)	1.0 (0.5 – 2.6)	0.5 (0.2 – 1.3)	<0.001
Time to first consultation >4h	134 (10.3)	57 (5.4)	<0.001
Pneumothorax	18 (1.4)	19 (1.8)	0.528
Cooling	17 (1.3)	14 (1.3)	0.961
Home oxygen	129 (9.9)	146 (13.8)	<0.001
Mortality	12 (0.9)	1 (0.1)	<0.001
Length of stay (days)	4 (2 – 11)	2 (1 – 4)	<.0.001
Postmenstrual age at discharge (wks)	38.1 (36.0 – 40.2)	39.1 (36.5 – 40.4)	<0.001
Weight at discharge	2.66 (2.04 – 3.39)	3.08 (2.42 – 3.58)	<0.001

Comparisons by Mann-Whitney U or Chi square test as appropriate.

Confidential: For Review Only

Following binary regression analysis, the highest quintile of the length of stay (10-89 days) was independently associated with the admission year (Odds ratio: 0.90, 95% CI: 0.86-0.93, adjusted p<0.001), gestational age (Odds ratio: 0.72, 95%CI: 0.69-0.75, adjusted p<0.001), birth weight z-score (Odds ratio: 0.71, 95% CI: 0.63-0.81, adjusted p<0.001), admission temperature (Odds ratio: 0.18, 95% CI: 1.33-2.31, adjusted p<0.001) and cord pH (Odds ratio: 17.75, 95% CI: 5.09-61.81, adjusted p<0.001). Cox proportional hazards analysis demonstrated that the admission year was significantly associated with the length of stay (Hazard ratio: 1.04, 95% CI: 1.03-1.05, adjusted p<0.001) after correcting for gestational age (Hazard ratio: 1.17, 95% CI: 1.16-1.18, adjusted p<0.001).

DISCUSSION

We have demonstrated that cross-site working between a level three and a level one neonatal unit was associated with improved short-term neonatal outcomes including less admission hypothermia, shorter duration of stay and more timely first consultation by senior members of the team.

Our results highlight a successful method in which a level-one unit can maintain and improve quality standards by working together with a tertiary unit. This has implications within networks of neonatal care, as such models could be adopted more widely to improve the care of infants born in hospitals without tertiary neonatal facilities. Of note, the results of our study relate to a unit with a workload which is higher compared to other level one units in London in the same study periods. A recent survey of neonatal units reported live births ranging from two to three thousands per year in other level one units in London [14]. Our method thus could be feasible in the lower-volume level one units.

We could not compare our study results with studies reporting on interventions to improve neonatal outcomes on level one units, as to the best of our knowledge there are no other such studies in the literature. Cross-site working between a level three and a level one unit is

https://mc.manuscriptcentral.com/bmjpo

Confidential: For Review Only

not the only potential model of care that could theoretically improve neonatal outcomes in level one units. Other described models include the development of the roles of the enhanced or advanced neonatal nurse practitioners, physician associates or associate specialists [14]. These, however, are mostly recent developments with a paucity of published evidence on the impact of these models on neonatal outcomes. A number of other factors can affect neonatal outcomes such as the impact of culture and morale on the workforce, investment in clinical supervision and training, implementation of quality improvement projects and integrated family development care. We could not capture these outcomes, as they are not quantified and documented in the neonatal data entry software [14].

Some of the improved outcomes that we have reported might also be explained by improved maternity care which might be the product of cross-site working in the maternity services. Such improved outcomes include a smaller percentage of infants born with a low cord pH and a lower number of infants born at less than 32 weeks of gestation, signalling more efficient in utero transfers of women at risk. The consistent presence of neonatal Consultants who did not have commitments to other paediatric services facilitated better and more frequent communication and interaction between the neonatal team and the obstetric and midwifery teams. Furthermore, improved admission temperature might also be related to better maternity care in the delivery suite, obstetric theatres and postnatal wards. The non-significant increase in the administration of antenatal steroids might be explained by the smaller percentage of infants that delivered prematurely in the PRUH and would thus qualify for this intervention [15]. The smaller percentage of infants that had a lower Apgar score at 10 minutes might be related to improved resuscitation at birth, but could also be partly explained by the smaller number of infants that were born with a lower cord arterial pH. We recognise, thus, that maternity improvement clearly played a major part in our improved neonatal outcomes. The majority, though, of the reported outcomes in our study are direct quality indicators of neonatal care and could be partly attributed to improved multi-professional neonatal care [1]. We should also note that the smaller percentage of infants

Confidential: For Review Only

that delivered below 32 weeks of gestation in the cross-site period might have contributed to some improved outcomes that are more relevant to preterm infants such as admission hypothermia and a decreased length of stay.

Our study has significant financial implications. Neonatal services are costly as they require expensive equipment, consumables and employment of a skilled multi-professional workforce which operates 24 hours a day. Neonatal units have been reported to have an incomplete understanding of the costs of running the service [16]. We did not perform a separate financial analysis, as all care days were at the same level and cost. We reported, however, that the median length of stay was halved, which corresponds to a cost reduction of a similar magnitude. It follows that if this intervention was implemented on a wider scale, it could be associated with significant cost reductions. Daily charges for neonatal care vary across different Trusts, with little consensus on the basis on which these charges are determined, but an average cost of special care per day is approximately 500 GBP [17]. At a single level one unit, thus, with 400 admissions per year the cost saving would be approximately 100,000 GBP per year. We do not present in this paper a full financial analysis of the intervention but these numbers should be taken in the context of the relevant staffing and equipment expenditure. Irrespective of the financial aspects, it is important to remember that decreased length of stay and earlier discharge by senior clinicians promote normal family life in a home environment with enhanced quality of sleep, less exposure to infection risks [18] and improved neurodevelopment [19].

Interestingly, the introduction of a cross-site model was not associated with a reduction of the total number of admissions per year. This might be explained by the fact that newborn infants constitute an already high-risk patient population. According to the World Health Organisation in 2019, 47% of all deaths in under five year olds occurred in the newborn period [20]. Previous neonatal studies have focused on improving outcomes of premature infants and more so the extremely premature ones. It is interesting, however, to note that the vast majority of births are term or late preterm and neonatal admissions are commonly

https://mc.manuscriptcentral.com/bmjpo

Confidential: For Review Only

infants who require a brief episode of care. Our study is thus relevant to a wider population than the results of the specialised studies of extreme prematurity and more applicable to the wider public.

Our study has strengths and some limitations. We used a large population of infants that were cared for on the same unit minimising potential differences that relate to different clinical practices or standards of care between units. The applicability of our study lies in that the improved short-term neonatal outcomes following the introduction of cross-site working, if confirmed by larger and more diverse-population studies, could strengthen the argument of implementing a cross-site neonatal model of care. We acknowledge as a limitation that it is impossible to completely separate the relative contribution of cross-site working to improved outcomes from the overall global tendency for better outcomes in neonatal care. For example, there has been an international drive to recognise and prevent neonatal hypothermia following the recognition of the detrimental effects of this complication on neonatal outcomes [21]. The International Liaison Committee on Resuscitation recommended in a consensus statement in 2015 that the admission temperature of newborn infants should be maintained between 36.5 and 37.5 ^{o}C after birth, during stabilisation and admission, as temperature was a strong predictor of mortality and morbidity [22]. Other outcomes though, such as earlier consultation and discharge and timely in utero transfer, can be predominantly attributed to the implementation of novel workforce models such as cross-site working. Our study cannot establish a clear causal link between the introduction of cross-site working and improved neonatal outcomes as it is retrospective and spans over twelve years. Nevertheless, it is important to share with the neonatal community our experience of this intervention which could have significant policy implications.

In conclusion, cross-site working between a level three and a level one neonatal unit was associated with improved short-term neonatal outcomes relevant to level one neonatal care, such as a reduction in admission hypothermia, shorter length of stay and more timely first consultation by senior members of the team.

Confidential: For Review Only

Legend to figures

Figure 1: Evolution of the length of stay (a), time to first consultation (b) and admission temperature (c) over the study period. The horizontal lines in the boxes represent the lower quartile, median and upper quartile values of the presented parameters and the whiskers the minimum and maximum values. The whiskers do not include the outliers, defined as values more than one and a half box lengths from the median which are presented as open circles. The whiskers do not include the extreme outliers, defined as values more than three box lengths from the median which are presented as asterisks.

Confidential: For Review Only

Funding statement: This project was partially supported by King's College London 2018 Medical Research Council Confidence in Concept Award through the King's Health Partners' Research and Development Challenge Fund.

Competing interests statement: The authors have no competing interests to declare.

Author's contribution: TD conceived the study, contributed to data analysis and interpretation and wrote the first version of the manuscript. LS collected the data and contributed to data analysis and writing the manuscript. AH led the implementation of the intervention, contributed to study design and critically revised the manuscript. ES lead the implementation of the intervention and critically revised the manuscript. LL contributed to the study design and critically revised the manuscript. LP contributed to the study design and critically revised the manuscript. VWM contributed to the study design and critically revised the manuscript. RB led the implementation of the intervention, contributed to study design and critically revised the manuscript. AG contributed to project supervision, funding acquisition and extensively revised and edited the manuscript. All authors have read and agreed to the published version of the manuscript.

Data sharing statement: Data is available to share upon request.

Confidential: For Review Only

References

1. NHS. Toolkit for High Quality Neonatal Services. London, UK2009.

2. Improvement NEaN. Implementing the Recommendations of the Neonatal Critical Care Transformation Review. In: Improvement NEaN, editor. 2019.

3. BAPM. Service standards for hospitals providing neonatal care. 3rd Edition. 2010.

4. Luu TM, Rehman Mian MO, Nuyt AM. Long-Term Impact of Preterm Birth: Neurodevelopmental and Physical Health Outcomes. Clinics in perinatology. 2017 Jun;44(2):305-14. PubMed PMID: 28477662. Epub 2017/05/10.

5. Helenius K, Longford N, Lehtonen L, Modi N, Gale C, Neonatal Data Analysis U, et al. Association of early postnatal transfer and birth outside a tertiary hospital with mortality and severe brain injury in extremely preterm infants: observational cohort study with propensity score matching. Bmj. 2019 Oct 16;367:l5678. PubMed PMID: 31619384. Pubmed Central PMCID: PMC6812621 www.icmje.org/coi_disclosure.pdf and declare: no support from any organisation for the submitted work; no financial relationships with any organisations that might have an interest in the submitted work in the previous three years; no other relationships or activities that could appear to have influenced the submitted work. Epub 2019/10/18.

6. Boyle EM, Johnson S, Manktelow B, Seaton SE, Draper ES, Smith LK, et al. Neonatal outcomes and delivery of care for infants born late preterm or moderately preterm: a prospective population-based study. Archives of disease in childhood Fetal and neonatal edition. 2015 Nov;100(6):F479-85. PubMed PMID: 25834169. Pubmed Central PMCID: PMC4680176. Epub 2015/04/03.

7. Suff N, Story L, Shennan A. The prediction of preterm delivery: What is new? Seminars in fetal & neonatal medicine. 2019 Feb;24(1):27-32. PubMed PMID: 30337215. Epub 2018/10/20.

Confidential: For Review Only

8 https://www.ons.gov.uk/aboutus/transparencyandgovernance/freedomofinformationfoi/hospitalstatistics: Office for National Statistics; [cited 2022 03/05].

9. Smith LK, Draper ES, Manktelow BN, Field DJ. Socioeconomic inequalities in survival and provision of neonatal care: population based study of very preterm infants. Bmj. 2009 Dec 1;339:b4702. PubMed PMID: 19952036. Pubmed Central PMCID: PMC2786957. Epub 2009/12/03.

10. Yeh P, Emary K, Impey L. The relationship between umbilical cord arterial pH and serious adverse neonatal outcome: analysis of 51,519 consecutive validated samples. BJOG. 2012 Jun;119(7):824-31. PubMed PMID: 22571747. Epub 2012/05/11.

11. Wright CM, Booth IW, Buckler JM, Cameron N, Cole TJ, Healy MJ, et al. Growth reference charts for use in the United Kingdom. Archives of disease in childhood. 2002 Jan;86(1):11-4. PubMed PMID: 11806873. Pubmed Central PMCID: PMC1719041. Epub 2002/01/25.

12. Laptook AR, Bell EF, Shankaran S, Boghossian NS, Wyckoff MH, Kandefer S, et al. Admission Temperature and Associated Mortality and Morbidity among Moderately and Extremely Preterm Infants. The Journal of pediatrics. 2018 Jan;192:53-9 e2. PubMed PMID: 29246358. Pubmed Central PMCID: PMC5808888. Epub 2017/12/17.

13. BAPM. Identification and Management of Neonatal Hypoglycaemia in the Full Term Infant. 2017.

14. Mitra T, Bramwells L. Exploring New Ways of Working in the Neonatal Unit. London: London Neonatal Operational Delivery Network; 2017.

15. Chawla S, Natarajan G, Shankaran S, Pappas A, Stoll BJ, Carlo WA, et al. Association of Neurodevelopmental Outcomes and Neonatal Morbidities of Extremely Premature Infants With Differential Exposure to Antenatal Steroids. JAMA pediatrics. 2016 Dec 1;170(12):1164-72. PubMed PMID: 27723868. Pubmed Central PMCID: PMC5294968. Epub 2016/10/11.

Confidential: For Review Only

16. Accounts HoCCoP. Caring for Vulnerable Babies: The reorganisation of neonatal services in England. London House of Commons; 2008.

17. Dassios T, Greenough A, Leontiadi S, Hickey A, Kametas NA. Admissions for hypoglycaemia after 35 weeks of gestation: perinatal predictors of cost of stay. The journal of maternal-fetal & neonatal medicine : the official journal of the European Association of Perinatal Medicine, the Federation of Asia and Oceania Perinatal Societies, the International Society of Perinatal Obstet. 2019 Feb;32(3):448-54. PubMed PMID: 28922987. Epub 2017/09/20.

18. Jones E, Stewart F, Taylor B, Davis PG, Brown SJ. Early postnatal discharge from hospital for healthy mothers and term infants. The Cochrane database of systematic reviews. 2021 Jun 8;6:CD002958. PubMed PMID: 34100558. Pubmed Central PMCID: PMC8185906. Epub 2021/06/09.

19. Losbar JL, Montjaux N, Ehlinger V, Germany L, Arnaud C, Tscherning C. Early discharge and hospital-assisted home care is associated with better neurodevelopmental outcome in preterm infants. Early human development. 2021 Oct;161:105451. PubMed PMID: 34507020. Epub 2021/09/11.

20. WHO. Newborns: improving survival and well-being: WHO 2020 [cited 2022 02/05]. Available from: https://www.who.int/news-room/fact-sheets/detail/newborns-reducing-mortality#:~:text=Causes,within%20the%20first%2024%20hours.

21. Lunze K, Bloom DE, Jamison DT, Hamer DH. The global burden of neonatal hypothermia: systematic review of a major challenge for newborn survival. BMC medicine. 2013 Jan 31;11:24. PubMed PMID: 23369256. Pubmed Central PMCID: PMC3606398. Epub 2013/02/02.

22. Perlman JM, Wyllie J, Kattwinkel J, Wyckoff MH, Aziz K, Guinsburg R, et al. Part 7: Neonatal Resuscitation: 2015 International Consensus on Cardiopulmonary Resuscitation and Emergency Cardiovascular Care Science With Treatment Recommendations. Circulation. 2015 Oct 20;132(16 Suppl 1):S204-41. PubMed PMID: 26472855. Epub 2015/10/17.

Confidential: For Review Only

Confidential: For Review Only

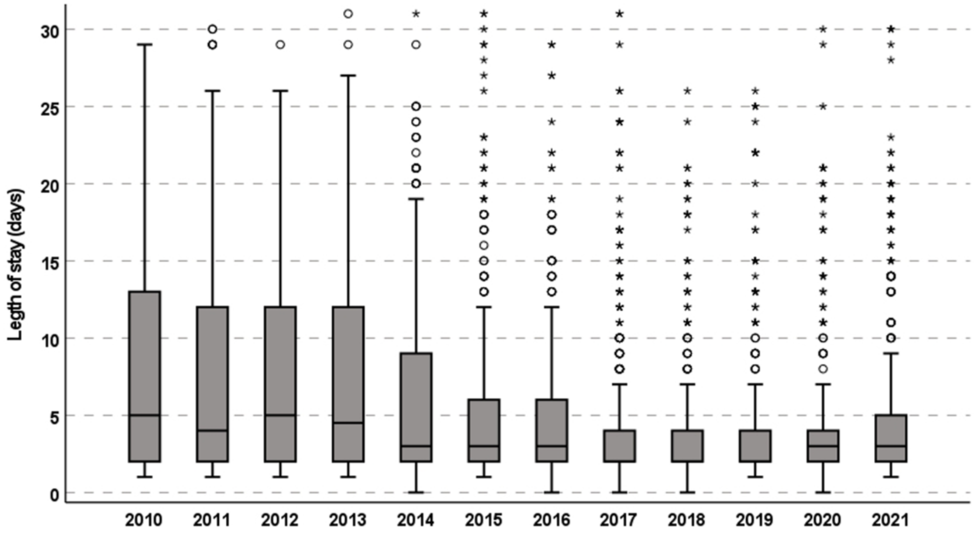

Figure 1: Evolution of the length of stay (a), time to first consultation (b) and admission temperature (c) over the study period. The horizontal lines in the boxes represent the lower quartile, median and upper quartile values of the presented parameters and the whiskers the minimum and maximum values. The whiskers do not include the outliers, defined as values more than one and a half box lengths from the median which are presented as open circles. The whiskers do not include the extreme outliers, defined as values more than three box lengths from the median which are presented as asterisks.

300x177mm (300 x 300 DPI)

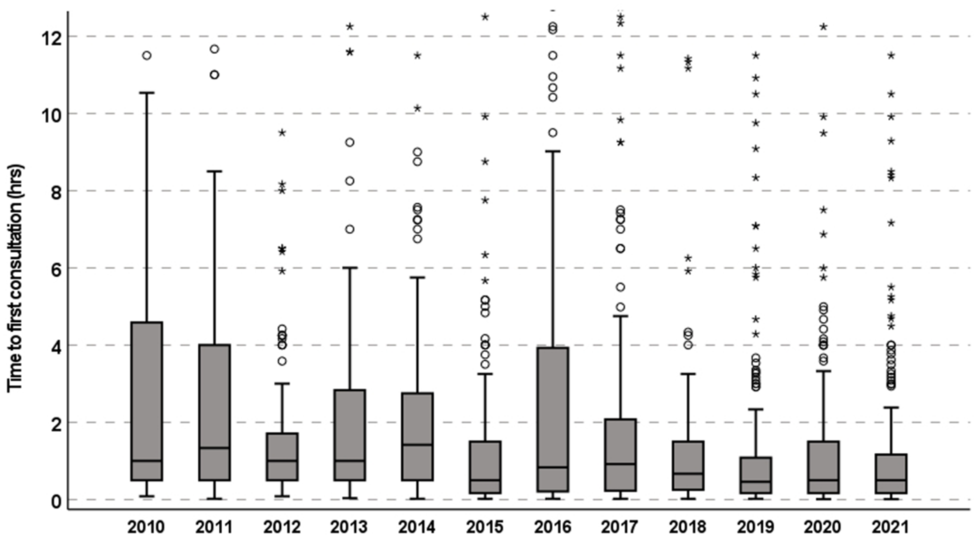

Figure 1: Evolution of the length of stay (a), time to first consultation (b) and admission temperature (c) over the study period. The horizontal lines in the boxes represent the lower quartile, median and upper quartile values of the presented parameters and the whiskers the minimum and maximum values. The whiskers do not include the outliers, defined as values more than one and a half box lengths from the median which are presented as open circles. The whiskers do not include the extreme outliers, defined as values more than three box lengths from the median which are presented as asterisks.

300x177mm (300 x 300 DPI)

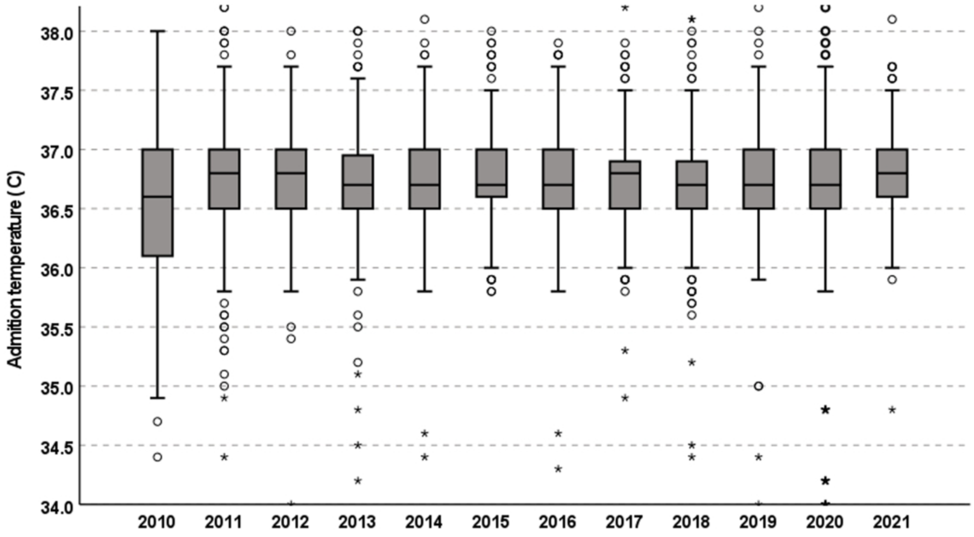

Figure 1: Evolution of the length of stay (a), time to first consultation (b) and admission temperature (c) over the study period. The horizontal lines in the boxes represent the lower quartile, median and upper quartile values of the presented parameters and the whiskers the minimum and maximum values. The whiskers do not include the outliers, defined as values more than one and a half box lengths from the median which are presented as open circles. The whiskers do not include the extreme outliers, defined as values more than three box lengths from the median which are presented as asterisks.

300x177mm (300 x 300 DPI)

Go straight to content.

Medical Research Council

NHS Health Research Authority

Is my study research?

To print your result with title and IRAS Project ID please enter your details below:

Title of your research:

Multi-professional cross-site working between a level one and a level three neonatal unit: Impact on clinical outcomes

Corresponding author:
Dr Theodore Dassios, PhD, FRCPCH
Reader in Neonatology, School of Life Course Sciences, King's College London,
Consultant Neonatologist, King's College Hospital NHS Foundation Trust,
Neonatal Unit, Golden Jubilee Wing, King's College Hospital,
Denmark Hill, SE5 9RS, London, UK

IRAS Project ID (if available): Not applicable

You selected:

- **'No'** - Are the participants in your study randomised to different groups?
- **'No'** - Does your study protocol demand changing treatment/ patient care from accepted standards for any of the patients involved?
- **'No'** - Are your findings going to be generalisable?

Your study would NOT be considered Research by the NHS.

You may still need other approvals.

Researchers requiring further advice (e.g. those not confident with the outcome of this tool) should contact their R&D office or sponsor in the first instance, or the HRA to discuss your study. If contacting the HRA for advice, do this by sending an outline of the project (maximum one page), summarising its purpose, methodology, type of participant and planned location as well as a copy of this results page and a summary of the aspects of the decision(s) that you need further advice on to the HRA Queries Line at Queries@hra.nhs.uk.

For more information please visit the Defining Research table.

Follow this link to start again.

Print This Page

NOTE: If using Internet Explorer please use browser print function.

About this tool Feedback Contact Glossary Accessibility
